# A secreted protein of *Mycoplasmopsis synoviae*, SSB, is a critical adhesion factor that promotes the enrichment of Annexin A2 at the cell membrane

**DOI:** 10.1128/spectrum.00858-26

**Published:** 2026-08-27

**Authors:** Qing Wang, Yunhai Zhao, Haiyun Ma, Xiaoxiao He, Yuting Zhang, Xiaoyong Xing, Guomei Quan, Duoduo Si, Zhixiong Zhang, Shijun Bao, Xiaochun Wu

**Affiliations:** 1 College of Veterinary Medicine, Gansu Agricultural University 74661, Lanzhou, Gansu, China; Griffith University - Gold Coast Campus, Gold Coast, Australia; Huazhong Agricultural University, Wuhan, China

**Keywords:** *M. synoviae*, secreted protein, SSB, adhesion, Annexin A2, colocalization

## Abstract

*Mycoplasmopsis synoviae* is an important pathogen in poultry that primarily causes synovitis, tenosynovitis, eggshell abnormalities, and respiratory diseases, resulting in significant economic losses to the poultry industry worldwide. The aim of this study was to systematically screen and characterize the proteins secreted by *M. synoviae* and to investigate the role of SSB in *M. synoviae* infection. We first identified 13 proteins secreted by *M. synoviae* using liquid chromatography‒tandem mass spectrometry analysis. Bioinformatic analysis of SSBs revealed that they lack a transmembrane domain and the characteristic features of a cleavable signal peptide and are potential virulence factors. After induced expression and purification of SSB from *Escherichia coli*, rabbit anti-SSB serum was prepared. Subcellular localization analysis by immunogold labeling and Western blotting further confirmed that SSB is present mainly in the cytoplasm and culture supernatant of *M. synoviae*. ELISA and indirect immunofluorescence analyses revealed that SSB binds to membrane proteins and adheres to the surface of chicken DF-1 cells and that rabbit anti-SSB serum significantly inhibits *M. synoviae* adhesion to DF-1 cells. *In vitro* experiments confirmed that SSB could interact directly with plasminogen and fibronectin in a dose-dependent manner. In addition, our study revealed that SSB significantly promoted Annexin A2 (AnxA2) protein expression in DF-1 cells during adhesion, significantly colocalized with AnxA2, and promoted its enrichment at the membrane. These findings offer a novel experimental foundation for understanding the molecular mechanisms underlying the role of the secreted protein SSB in *M. synoviae* infection.

**IMPORTANCE:**

Pathogenic bacteria transport proteins to the extracellular compartment through a variety of secretion systems, and most of these secreted proteins act as adhesins, toxins, or immunomodulatory molecules in host cells, playing an important role in bacterial pathogenesis. However, studies on secreted proteins of *Mycoplasmopsis synoviae* are still incomplete. In our study, we systematically screened and identified the secreted proteins of *M. synoviae* strain WVU1853 for the first time and conducted an in-depth investigation of the role of SSB, one of the secreted proteins, in the infection process, *in vitro*. Our results not only reveal the key role of SSB in the infection process of *M. synoviae* but also provide a new theoretical basis for studying the invasion mechanisms of *Mycoplasma*-secreted proteins and related processes.

## INTRODUCTION

*Mycoplasmopsis synoviae* belongs to the class *Mollicutes* and lacks a cell wall (1). It was first reported as an unknown pathogen in the United States in 1954 (2) and is one of the smallest self-replicating prokaryotic organisms found to date (3). Although they do not cause high mortality rates (4), *M. synoviae* infections in chickens and turkeys result in tenosynovitis, respiratory lesions, eggshell apex abnormalities, reduced egg production, and decreased carcass weight in poultry (5, 6), causing significant economic losses in the poultry industry. Notably, immunosuppression after *M. synoviae* infection can lead to coinfection with other avian pathogens, such as Newcastle disease virus, infectious bronchitis virus, and avian reovirus, which can significantly aggravate the disease and result in more serious economic losses (7, 8). Therefore, research on *M. synoviae* should lay a theoretical foundation for a deeper understanding of the pathogenesis of *M. synoviae* and the establishment of effective diagnostic methods, as well as preventive and control measures.

*Mycoplasma* establishes chronic infection in the host through multiple pathways, with adhesion being the first and most critical step in colonization and infection (9). Previous studies have shown that several *M. synoviae* proteins are involved in adhesion, such as PdhD (10), LP78 (11), EF-Ts (12), NOX (13), EF-G (14), PdhA, PdhB (15), and E2 (16), but these studies have focused primarily on the membrane proteins of *M. synoviae*. In recent years, secreted proteins have gained attention because they are intimately involved in a variety of functions, such as nutrient uptake, adhesion, and invasion (17). The proteins secreted by several mycoplasmas play important roles in pathogenicity. For example, in *Mycoplasma bovis*, MbovP280 induces apoptosis via its coiled-coil domain and the cellular ligand CRYAB (18), and the secreted nuclease MBOV-RS02825 degrades the DNA matrix of neutrophil extracellular traps and induces macrophage apoptosis (19). In addition, MbovP0725 inhibits the host inflammatory response by decreasing the phosphorylation level of the MAPK signaling pathway (20); on the other hand, Mpn491, a protein secreted by *Mycoplasmoides pneumoniae*, mediates pathogen evasion of the killing effect of neutrophil extracellular traps (21). These studies suggest that secreted proteins play a key role in mycoplasma infections.

Single-strand DNA-binding proteins (SSBs) are indispensable in DNA metabolism and can specifically bind to single-strand DNA regions to regulate important processes such as DNA replication, recombination, and repair (22). In recent years, it has been shown that mitochondrial single-stranded DNA-binding protein 1 (SSBP1) can increase p53 phosphorylation and its nuclear accumulation through interaction with DNA-dependent protein kinases, which subsequently drives ferroptosis in podocytes (23). Concurrently, studies have confirmed that SSB is one of the immunogenic proteins of *Brucella abortus*, capable of eliciting a specific reaction with *Brucella abortus*-positive sera, demonstrating potential for clinical diagnosis (24). In *Xanthomonas*, single-stranded DNA-binding protein (SSBx) is a secreted protein dependent on the type III secretion system that can elicit a hypersensitive response and programmed cell death (25). Recent research further indicates that a mutation at amino acid position C514 of the herpesvirus single-stranded DNA-binding protein, ICP8, reduces its ability to bind to single-stranded DNA, thereby attenuating the virulence of duck plague virus (DPV) while inducing a potent protective immune response in the host (26). This provides new targets and important experimental evidence for the development of safe, effective, and non-reverting DPV genetic engineering vaccines. However, most SSB-related studies have focused on eukaryotes, and there are no reports on SSB function in *M. synoviae*.

In this study, 13 proteins secreted by *M. synoviae* were identified using liquid chromatography‒tandem mass spectrometry (LC‒MS/MS) analysis. Among them, SSB, as a potential virulence factor, is distributed mainly in the cytoplasm and culture supernatants and interacts with plasminogen (Plg) and fibronectin (Fn), significantly adhering to chicken DF-1 cells and promoting Annexin A2 (AnxA2) enrichment at the cell membrane by colocalizing with AnxA2. These findings not only identify candidate proteins for the development of *M. synoviae* vaccines but also deepen our understanding of its pathogenic mechanisms.

## MATERIALS AND METHODS

### Bacterial strains, plasmids, and cell lines and cultivation

*M. synoviae* strain WVU1853 was isolated from chicken joints in the United States in 1957 (27). The strain was obtained from the China Veterinary Conservation Center (Beijing, China). It was cultured in mycoplasma broth base (Haibo, Shandong, China) supplemented with 10% swine serum (Hongquan, Guangzhou, China), 0.01% NAD (Roche Diagnostics, Basel, Switzerland), 0.01% L-cysteine (Solarbio, Beijing, China), and 80,000 IU of penicillin. All cultures were incubated at 37°C in a 5% CO_2_ incubator. The *Escherichia coli* strain BL21 (DE3) was purchased from TransGen Biotech Co., Ltd (Beijing, China). Chicken embryo fibroblast (DF-1) cells were obtained from Procell Biotech Co., Ltd. (Wuhan, China) and cultured in Dulbecco’s modified Eagle’s medium (GIBCO, Grand Island, NY, USA) supplemented with 10% fetal bovine serum (Biological Industries, Shanghai, China) and 1% penicillin–streptomycin liquid (Solarbio). ChamQ Universal SYBR qPCR Master Mix was purchased from Vazyme Biotech Co., Ltd. (Nanjing, China). The New Zealand white rabbits were purchased from the Animal Center of Lanzhou Veterinary Research Institute (Lanzhou, China). The polyclonal anti-AnxA2 serum was prepared in our laboratory.

### Enrichment of proteins from the *M. synoviae* culture supernatant

To obtain secreted proteins, 1,000 mL of *M. synoviae* was cultured until the logarithmic growth phase was reached after 36 h. The timing of the logarithmic growth phase was defined by the standard growth curve of the *M. synoviae* WVU1853 strain (28). The supernatant was collected by centrifugation at 12,000 × *g* for 45 min at 4°C (Beckman Coulter, Inc., Brea, CA, USA) to remove intact mycoplasma cells. Proteins in the supernatant were kept on ice throughout enrichment using a tangential flow filtration membrane package (Sartorius, Germany) with a molecular weight (MW) cutoff of 10 kDa at a pump speed of 60 rpm. After the supernatant was concentrated to approximately 50 mL, the retentate was then collected and dialyzed using PEG 6,000 (Biosharp, Anhui, China) with a dialysis cutoff of 3,000 Da (Solarbio) at 4°C to further concentrate it to a volume of 10 mL. All harvest samples were temporarily stored at −80°C.

### Screening and identification of the proteins secreted by *M. synoviae* using LC‒MS/MS

The identification of secreted proteins was completed by professionals using LC‒MS/MS (Thermo Fisher Scientific, Waltham, MA, USA). All related bioinformatics analyses were performed using a database from the National Center for Biotechnology Information (NCBI, NLM, NIH, Bethesda, MD, USA; accession no. CP011096.1) and commercial services provided by Shanghai OE Biotech Co., Ltd. (Shanghai, China). Amino acid sequence alignment was used to confirm protein homology using the NCBI database, and peptide sequence coverage was calculated from LC–MS/MS raw data. The above identification results were analyzed, and the SSB sequence was obtained from the NCBI database. The transmembrane domains, signal peptides, and virulence proteins of *M. synoviae* were predicted using the following websites: TMHMM Server (TMHMM 2.0, DTU Health Tech–Bioinformatic Services), SignalP-4.1 (SignalP 4.1, DTU Health Tech–Bioinformatic Services), and VirulentPred 2.0 (https://bioinfo.icgeb.res.in/virulent2/index.html).

### Sequence alignment, phylogenetic tree construction, and conserved domain analysis

Using the DNASTAR software (DNASTAR, Inc., Madison, WI, USA), the SSB amino acid sequences (AKB11260.1) of the *M. synoviae* WVU1853 strain were compared with those of *M. synoviae* HN01 (WP_154221707.1), *M. synoviae* H (AQU48076.1), *M. synoviae* 53 (WP_011283670.1), *M. synoviae* 5-9 (UBM43515.1), *M. synoviae* A4 (UBY00073.1), *M. synoviae* G3 (UBX99132.1), *Mycoplasma gallisepticum* (ADC30831.1), *M. pneumoniae* (XBW34012.1), *Mycoplasma mycoides* subsp. *mycoides* (AMK55955.1), *Mycoplasma agalactiae* (UUM25159.1), *Mycoplasma anserisalpingitidis* (QDY88381.1), *Mesomycoplasma hyorhinis* (QEA02011.1), *Mycoplasma hyopneumoniae* (WP_404924450.1), *M. hyopneumoniae* 7448 (AAZ53668.1), and *M. bovis* HB0801 (WP_013954802.1) from the NCBI database, and a homology heatmap was generated using GraphPad Prism 8.0 software (GraphPad Software, Inc., San Diego, CA, USA). Phylogenetic analysis was conducted using the MEGA 8.0 software (Institute of Molecular Evolutionary Genetics, Pennsylvania, USA). The phylogenetic tree was built via the neighbor-joining method with a Poisson correction model. Bootstrap analysis with 1,000 replicates was performed to evaluate the reliability of each branch. In addition, the SSB amino acid sequence was analyzed using the NCBI Conserved Domain Database (CDD). The predicted domain structure was then compared with the reported domain features of *E. coli* and *Bacillus subtilis* SSB from published literature (29, 30).

### SSB cloning, expression, and purification

The *ssb* sequence contains a TGA codon at 163–165 bp. This codon encodes tryptophan in mycoplasma, whereas in *E. coli*, it functions as a stop codon. To achieve site-directed codon mutations within the gene and successfully express SSB, overlap PCR was used to amplify the *ssb* and optimize the 165 bp site, and the *Bam*H I and *Xho* I enzyme restriction sites were added to the upstream and downstream primers, respectively. The primers used were synthesized by Tsingke (Beijing, China), and the specific sequences are shown in Table 1. The *ssb* and pET-28a (+) empty vectors were ligated using T4 DNA ligase (Vazyme Biotech Co., Ltd.). After sequence verification of a recombinant clone, the plasmid was transformed into *E. coli* BL21 (DE3), and protein expression was induced using the addition of 1 mM isopropyl β-D-thiogalactoside (Solarbio) at 16°C for 10 h. Cells were harvested by centrifugation at 8,000 × *g* for 10 min and washed three times with precooled PBS. The precipitates were resuspended in 10 mL of lysis buffer, followed by ultrasonication for 30 min under 200 W power. After centrifugation at 10,000 × *g* for 15 min, the supernatant was collected and purified from the supernatant using Ni-NTA His-Tag purification resin (MCE, Monmouth Junction, NJ, USA). The purified target protein was quantified and analyzed for purity using a BCA protein assay kit (Beyotime, Shanghai, China) and SDS‒PAGE, respectively.

**TABLE 1:** Primer and sequences used in the experiments^*a*^

Primer	Sequence (5′−3′)	Product length (bp)	Usage
*ssb*-F1	TTAGGATCCATGGCCGATTTAAATAAAGTAATT	173	PCR
*ssb*-R1	TGATTTCT***C***CAAGCTACAAA		
*ssb*-F2	TTTGTAGCTTG***G***AGAAATCA	414	PCR
*ssb*-R2	ACTCTCGAGTTAAAATCCTTTATCTAAATCTATCTATATCATCG		
AnxA2-F	GAGGCAGTGATCTTGGGCTT	88	RT‒qPCR
AnxA2-R	CATCAGTTCCCAGGCCCTTC		
GAPDH-F	CCCAGCAACATCAAATGGGC	115	RT‒qPCR
GAPDH-R	TAACACGCTTAGCACCACCC		
			

^*a*^ The restriction enzyme sites are underlined; the mutated site is in bold and italicized.

### Preparation of polyclonal anti-SSB serum

The purified product was used to immunize New Zealand white rabbits. The specific steps were as follows: prior to immunization, 1 mL of blood was collected from the auricular vein of the ears of New Zealand white rabbits as negative control sera. The purified protein was then mixed with an equal volume of Freund’s complete adjuvant (Sigma-Aldrich, St. Louis, MO, USA), fully emulsified, and injected subcutaneously at multiple points into rabbits at a dose of 800 μg. After 14 days, the immunization dose was halved a second time, and the mixture was emulsified with Freund’s incomplete adjuvant (Sigma-Aldrich). Subsequently, immunization was boosted every 7 days using the same dose and method as noted for the second immunization. On the fifth day after the last immunization, blood was collected from the heart, and the serum was separated.

### Subcellular localization of SSB

To further characterize the distribution of SSB, 100 mL of *M. synoviae* was cultured to the logarithmic stage. *M. synoviae* was collected by centrifugation at 8,000 × *g* at 4°C and rinsed with sterile PBS three times, after which the supernatant was discarded. Then, 4% paraformaldehyde (Biosharp) was added and resuspended, and the sample was fixed for 5 min at 4°C and then centrifuged at 12,000 × *g* at 4°C for 10 min. The supernatant was gently discarded, and the sediment was retained for immunoelectron microscopy analysis. Negative serum and anti-SSB serum were used as the primary antibodies, respectively.

One milliliter of *M. synoviae* culture was centrifuged at 8,000 × *g* for 10 min at 4°C and resuspended in PBS with 200 μL, and 30 μL of the mixture was collected, spread evenly on a slide, and left to dry naturally. The samples were fixed with 4% paraformaldehyde, incubated with primary antibodies (anti-SSB serum; preimmunization serum as a negative control and anti-*M*. *synoviae* serum as a positive control; all sera were diluted 1:1,000), incubated with goat antirabbit IgG/FITC antibodies (Bioss, Beijing, China) (dilution ratio of 1:500) as secondary antibodies, and finally observed under a fluorescence microscope (ZEISS, Germany).

The cell membrane and cytoplasmic proteins were separated using the Triton X-114 method as described previously with minor modifications as follows (12): *M. synoviae* was washed and resuspended in sterile PBS. Then, 1 mM protease inhibitor (Solarbio) and 4% Triton X-114 were added, and the mixture was incubated at 4°C for 30 min. The mixture was then incubated at 37°C for 10 min and centrifuged (Himac, Japan) at 12,000 × *g* for 20 min. The cytoplasmic protein fractions and membrane protein fractions were divided into upper aqueous layers and lower oil layers, respectively. The upper and lower liquid layers were collected separately and treated by adding four times the volume of precooled acetone at −20°C overnight. After that, the proteins were centrifuged at 12,000 × *g* for 15 min at 4°C, and an equal volume of 8 mol/L urea was dissolved in the membrane and cytoplasmic proteins. An equal volume of protein was used for Western blot analysis. Anti-SSB serum was used as the primary antibody, and goat antirabbit horseradish peroxidase (HRP)/IgG (Bioss) was used as the secondary antibody.

### Binding of SSB to chicken plasminogen (Plg) and fibronectin (Fn)

SSB was transferred to a polyvinylidene fluoride membrane in varying amounts (10–0.625 μg), and Plg or Fn was bound at 37°C for 2 h. The membrane was washed with phosphate-buffered saline with Tween-20 (PBST) to remove non-specific bound proteins, with each wash performed for 5 min and repeated five times. Anti-Plg and anti-Fn antibodies served as primary antibodies, and goat antirabbit IgG/HRP served as the secondary antibody. After each antibody incubation, the membrane was washed five times with PBST for 5 min. Enhanced chemiluminescence luminescent solution (ECL) (Oriscience, Chengdu, China) was used to visualize the protein bands. Moreover, 0.1 μg of Plg or Fn was added to each well. After the samples were blocked with 5% non-fat milk, SSB (50–0.39 μg) was added to each well, using bovine serum albumin (BSA) as a negative control. Anti-SSB serum was used as the primary antibody, and goat antirabbit IgG/HRP (1:5,000) was used as the secondary antibody. SSB binding to Plg and Fn was assessed by using optical density (OD_450_) absorbance measurements, and three independent replicates were performed.

### Adherence assay

The cells were fixed when DF-1 was cultured to 70% confluence in six-well plates, and 20 μg of SSB was added and incubated for 2 h at 37°C. After washing off non-specifically bound SSB with PBS, cells were fixed with 4% paraformaldehyde at room temperature for 20 min. The 6× His peptide (Beyotime) was used as a negative control. The preimmune serum and anti-SSB serum were diluted at a ratio of 1:2,000 for use as the primary antibody, and goat antirabbit IgG/FITC was used as the secondary antibody. The nuclei and membranes of the cells were stained with DAPI (Beyotime) and DIL (Beyotime), respectively, followed by washing with PBST to remove unbound dyes. Finally, the cells were observed with a DeltaVision Ultra Acquire Ultra Microscope (Cytiva, Marlborough, MA, USA).

The ability of SSB to bind to the cell membrane was detected using ELISA. The DF-1 membrane was extracted with a cell membrane protein extraction kit (Solarbio). The specific procedure was as follows: each well was coated with 0.5 μg of membrane proteins, and after blocking with 5% non-fat milk, different amounts of SSB (ranging from 50 to 0.39 μg) were added to bind to them (for the binding inhibition assay). The anti-SSB serum was diluted at a ratio of 1:50 in advance and incubated at 37°C for 2 h after mixing with SSB. The anti-SSB serum and goat antirabbit IgG/HRP were used as primary and secondary antibodies, with incubation at room temperature for 2 and 1.5 h, respectively. After washing off unbound antibodies with PBST, avoiding light, 3,3′,5,5′-tetramethylbenzidine color development solution was added. Then, 2 mol/L H_2_SO_4_ was added after 15 min, and the absorbance was measured at 450 nm. BSA was used as a negative control, and all assays were performed in three independent replicates.

### Adhesion inhibition assay of anti-SSB serum

The adhesion method was adapted slightly from existing protocols. Anti-*M*. *bovis* GrpE (Western blotting confirmed no cross-reactivity of this antibody against *M. synoviae*; refer to Fig. S2), preimmune serum, anti-SSB, and anti-*M*. *synoviae* sera were diluted (1:50) and preincubated with *M. synoviae* at 37°C for 1 h. The mixture was then added to DF-1 cells incubated for another 2 h, with gentle shaking every 30 min during incubation. After non-adhering *M. synoviae* were washed with PBS, the cells were fixed, stained, and observed for adhesion.

The effect of anti-SSB serum on adhesion was assessed by colony counting. Postinteraction, unadhered *M. synoviae* were removed, and the cells were collected via trypsin digestion, serially diluted 10-fold, and plated on *M. synoviae* agar. The plates were incubated at 37°C with 5% CO_2_ for 3‒5 days. Each dilution was tested in triplicate. Adhesion inhibition was calculated as follows: adhesion inhibition rate = (control group colonies − experimental group colonies) / control group colonies × 100%.

### Expression analysis of AnxA2 during SSB adhesion

To measure AnxA2 protein levels in DF-1 cells after SSB treatment, cells treated with empty pET-28a (+) products were used as the negative control, and total mRNA was extracted using TRIzol reagent (Thermo Fisher Scientific). cDNA was synthesized through reverse transcription with the reagent kit (ABclonal, Wuhan, China), and specific primers for AnxA2 and GAPDH were used for RT‒qPCR detection (Table 1). The RT‒qPCR setup included a 20 μL reaction with SYBR Green Master Mix 10 μL, cDNA 1 μL, upstream and downstream primers 0.2 μL, respectively, and RNase-free water supplemented up to 20 μL. qPCR was performed on a LightCycler 96 real-time PCR system (Roche) using SYBR Green qPCR Master Mix. The thermal cycling program was set as follows: initial denaturation at 95°C for 30 s, followed by 40 cycles of denaturation at 95°C for 10 s and combined annealing-extension at 60°C for 30 s. All RT‒qPCR assays were performed with three independent biological replicates, and each biological replicate contained three technical replicates. AnxA2 expression was calculated using the 2^−ΔΔCt^ method. Additionally, total protein was extracted from infected cells with RIPA lysis buffer (Solarbio), quantified by the BCA method, and analyzed via Western blotting. The protein bands were visualized using ECL and quantified with ImageJ software (National Institutes of Health, Bethesda, MD, USA).

### Colocalization of SSB with AnxA2

To test the interaction potential of SSB with AnxA2, DF-1 cells were inoculated in six-well plates containing round coverslips, and after the cell confluence reached 80%, the DF-1 cells were incubated with SSB at 39°C for 3 h. The cells were treated with the empty pET-28a (+) product as the negative control. The non-specific binding proteins were washed with PBS before fixation with DF-1 cells. IgG/Cy3 and IgG/FITC antibodies were used for double fluorescence staining, respectively. The nuclei were stained with DAPI and then imaged and analyzed using an Ultra Acquire Ultra Microscope for colocalization of SSB with AnxA2.

### Translocation detection of AnxA2

SSB and empty pET-28a (+) products were incubated with DF-1 cells for 9 h, respectively, before the DF-1 cell membrane and cytoplasmic proteins were extracted using the Cell Membrane Protein Extraction Kit (Beyotime). Changes in AnxA2 expression in the cell membrane and cytoplasm were detected by Western blotting with an anti-AnxA2 antibody (dilution ratio as 1:1,000) as the primary antibody. GAPDH (dilution ratio as 1:3,000) (Abmart, Shanghai, China) and Na^+^/K^+^-ATPase (1:10,000) (Selleck Chemicals, Houston, TX, USA) were used as labeling proteins for the cytoplasm and membrane, respectively. To further characterize the changes in the subcellular localization of AnxA2, goat antimouse IgG/Cy3 (Servicebio, Wuhan, China) was used as the secondary antibody, and AnxA2 translocation in DF-1 cells was observed using an Ultra Acquire Ultra Microscope.

### Statistical analysis

All the data are expressed as the means ± standard deviation. Western blot grayscale analysis was analyzed using ImageJ software. Statistical analysis of the experimental data were performed using GraphPad Prism 8.0 software. Differences between two groups were analyzed using a *t*-test (ns, no significant difference, *P* ≥ 0.05; *P < 0.05; **P < 0.01; ***P < 0.001; and ****P < 0.0001).

## RESULTS

### Thirteen proteins secreted by *M. synoviae* and bioinformatics analysis of SSB

To systematically identify the proteins secreted by *M. synoviae*, we analyzed the concentrated supernatant of *M. synoviae* in the logarithmic growth phase using LC‒MS/MS. A total of 13 *M. synoviae*-secreted proteins were identified. We further compared these 13 candidate proteins with homologs reported in another strain (31), and the detailed information, including sequence coverage, is listed in Table 2.

**TABLE 2:** Secreted proteins localized to the supernatant^*a*^^,^^*b*^

Accession no.	Protein names	Sequence coverage	Homologous protein in reference 31	Prediction results
AKB11101.1	Deoxyribonuclease	15	Not identified	Virulent
AKB11382.1	Elongation factor Tu	11	99.75%	Non-virulent
AKB11096.1	VY93_01090 rRNA methyltransferase	10	Not identified	Virulent
AKB10884.1	ABC transporter substrate-binding protein	9	Not identified	Virulent
AKB11260.1	Single-stranded DNA-binding protein	8	100%	Virulent
AKB11161.1	DNA helicase	8	Not identified	Virulent
AKB11174.1	Histidyl-tRNA synthetase	7	Not identified	Non-virulent
AKB10936.1	Hypothetical protein VY93_00850	6	Not identified	Virulent
AKB10998.1	Lipoprotein	5	Not identified	Virulent
AKB10971.1	Peptide ABC transporter ATP-binding protein	5	Not identified	Non-virulent
AKB10972.1	Hypothetical protein VY93_01090	2	Not identified	Virulent
AKB11015.1	Lipoprotein	2	Not identified	Virulent
AKB11330.1	ABC transporter permease	1	Not identified	Virulent

^*a*^ Acession number of proteins and protein names are derived from the NCBI database.

^*b*^ Not identified: it means that this protein was not detected in the proteominc dataset of the other strain in reference 31.

The prediction analyses conducted using Signal IP-4.1 and TMHMM 2.0 indicated that the SSB sequence lacks the characteristic features of a cleavable signal peptide, as evidenced by a *D* score of 0.132 (refer to Fig. S3a), which is significantly below the threshold of 0.75. Additionally, the sequence does not contain a transmembrane structural domain, with the predicted number of transmembrane helices being zero (refer to Fig. S3b). Furthermore, the VirulentPred 2.0 prediction analysis identified SSB as a potential virulence-associated protein (refer to Fig. S3c), suggesting its possible involvement in the pathogenesis process of *M. synoviae*.

### Sequence similarity, phylogenetic analysis, and conserved domain identification

**Fig 1:**
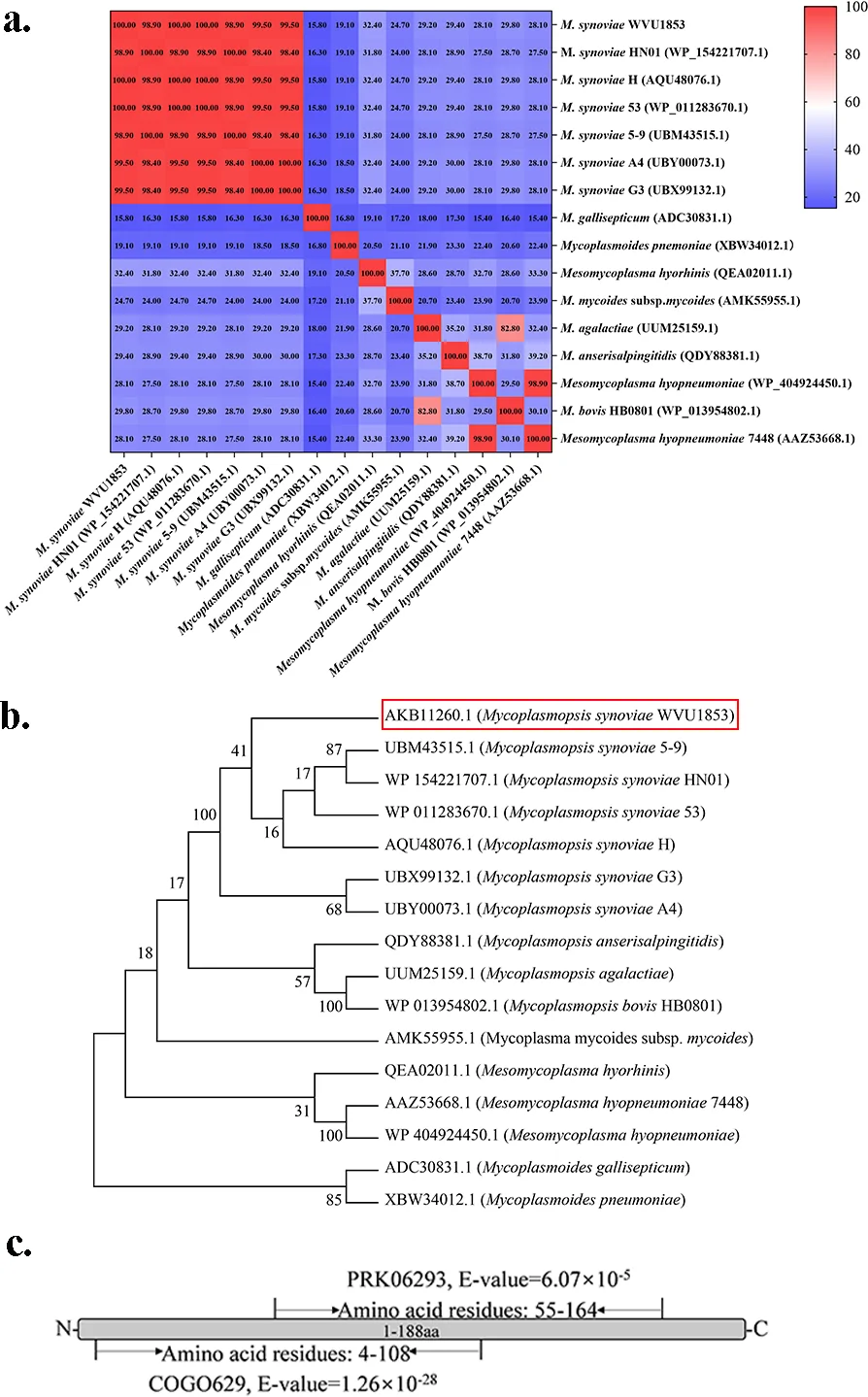
Homology analysis, phylogenetic tree of SSB, and conserved domain identification. (**a**) The homology heat map. (**b**) The phylogenetic tree was constructed based on multiple sequence alignment analysis. (**c**) Conserved domain identification of SSB.

Based on the homology heatmap and the phylogenetic tree, we analyzed the genetic and evolutionary relationships between *M. synoviae* strain WVU1853 and its closely related strains. Sequence homology analysis revealed that the SSB amino acid sequences of *M. synoviae* strain WVU1853 exhibited high homology (over 98%) with those of *M. synoviae* strains HN01, H, 53, A4, and G3. Homology with strains such as *M. gallisepticum* and *M. pneumoniae* was low (below 25%) (Fig. 1a). Phylogenetic tree analysis showed that *M. synoviae* WVU1853 clustered with strains HN01, H, 53, and 5-9 in the same evolutionary branch, exhibiting the closest phylogenetic relationship. It also showed a relatively high degree of relatedness with *M. synoviae* G3 and A4 while exhibiting the lowest relatedness with genera *Mesomycoplasma* and *Mycoplasmoides* (Fig. 1b). These findings indicate that SSB is highly conserved within the *M. synoviae* species, yet genetic differentiation still exists among different strains of the same species, while SSB sequences across genera show significant differentiation.

Conserved domain analysis via NCBI CDD revealed that the *M. synoviae* SSB contains only the canonical SSB domain. SSB consists of a core SSB domain (residues 4–108) and an overlapping extended SSB homology domain (residues 55–165) (Fig. 1c), both corresponding to the conserved core oligonucleotide/oligosaccharide-binding fold region, followed by a short C-terminal segment, consistent with the domain architecture of canonical SSB from *E. coli* and *Bacillus subtilis*. No additional functional domains were identified beyond the conserved C-terminal tail.

### Prokaryotic expression and secretion characterization of SSB

The overlap PCR product results demonstrated that the amplified specific bands corresponded to the anticipated 567 bp, confirming the successful construction of the prokaryotic expression vector pET-*ssb* of the *E. coli* (refer to Fig. S4a and b). According to the UniProt database, the theoretical MW of native SSB is 21.2 kDa. After fusing with the 6× His tag, the predicted MW of the recombinant SSB is approximately 25 kDa. Furthermore, SDS‒PAGE analysis of the expression products from *E. coli* BL21(DE3) revealed a distinct band at 25 kDa in the whole-cell lysate, supernatant, and precipitate, consistent with the theoretical MW of the recombinant protein. This band was absent in the control group transformed with the empty vector pET-28a (+), indicating the successful expression of the recombinant protein SSB (refer to Fig. S4c).

**Fig 2:**
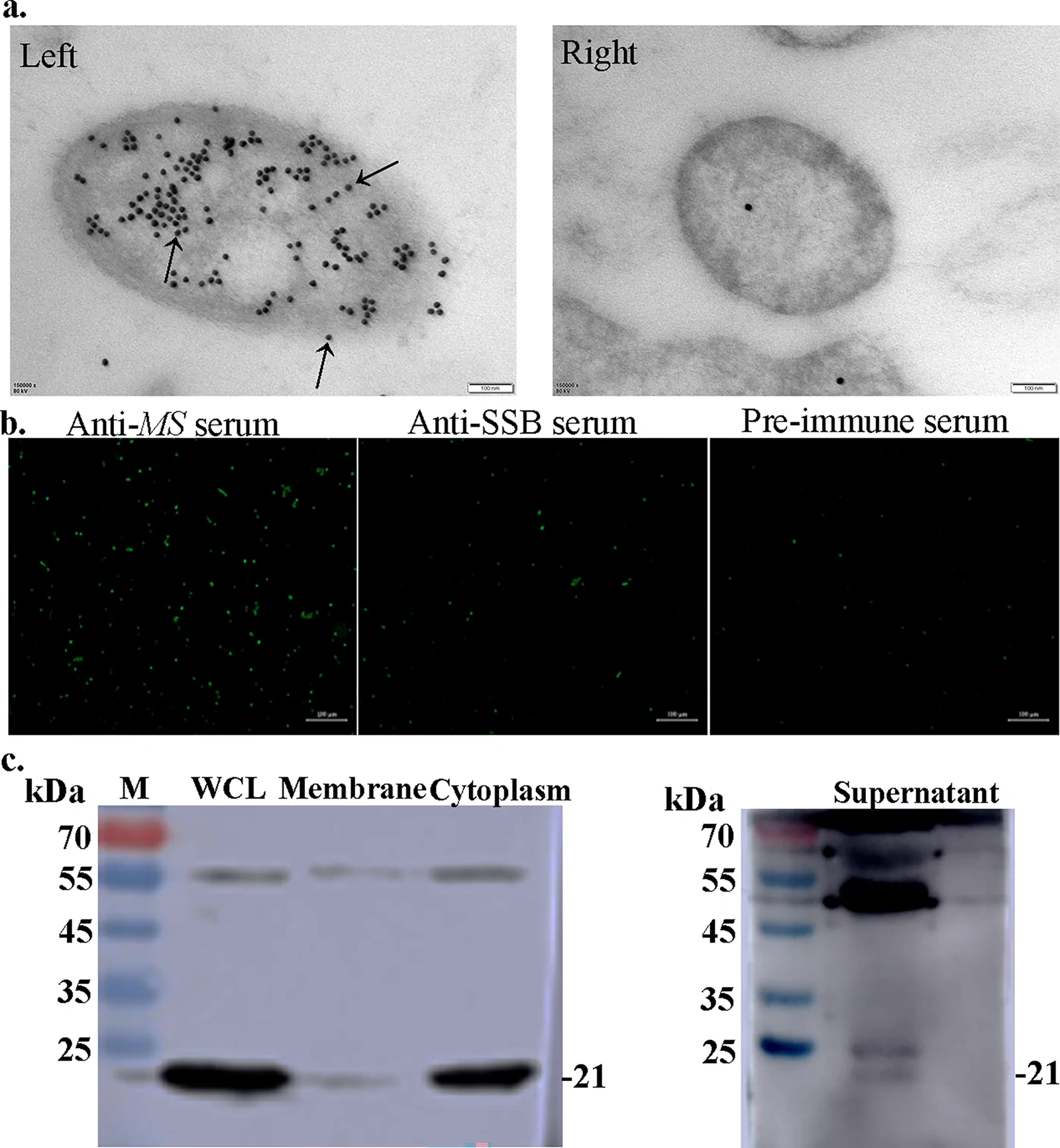
Subcellular localization of SSB in *M. synoviae*. (**a**) Immunogold labeling assay analysis of the distribution of SSB in *M. synoviae*. (Left) Anti-SSB serum was used as the primary antibody; (right) preimmune serum was used as the primary antibody. (**b**) Immunofluorescence tests were used to analyze the distribution of SSB, anti-*Mycoplasmopsis synoviae*, anti-SSB, and preimmune serum as primary antibodies, and goat antirabbit IgG/FITC was used as a secondary antibody. (**c**) Distribution of SSB in *M. synoviae* as demonstrated using Western blot analysis. Lane M, prestained protein marker; lane WCL, *M. synoviae* total protein; lane membrane, *M. synoviae* membrane protein; lane cytoplasm, *M. synoviae* cytoplasmic protein; lane supernatant: *M. synoviae* culture supernatant.

Immunogold labeling assays and immunofluorescence experiments demonstrated that SSB likely localizes exclusively within the cytoplasm of *M. synoviae* (Fig. 2a and b). To further elucidate the potential role of SSB as a secretory protein, Western blot analyses revealed that anti-SSB serum exhibited specific reactivity with *M. synoviae* lysates, cytoplasmic proteins, and supernatants, whereas it showed no reactivity with membrane proteins. These findings suggest that SSB is present in the cytoplasm and may be secreted extracellularly (Fig. 2c).

### SSB interacts with Plg and Fn

**Fig 3:**
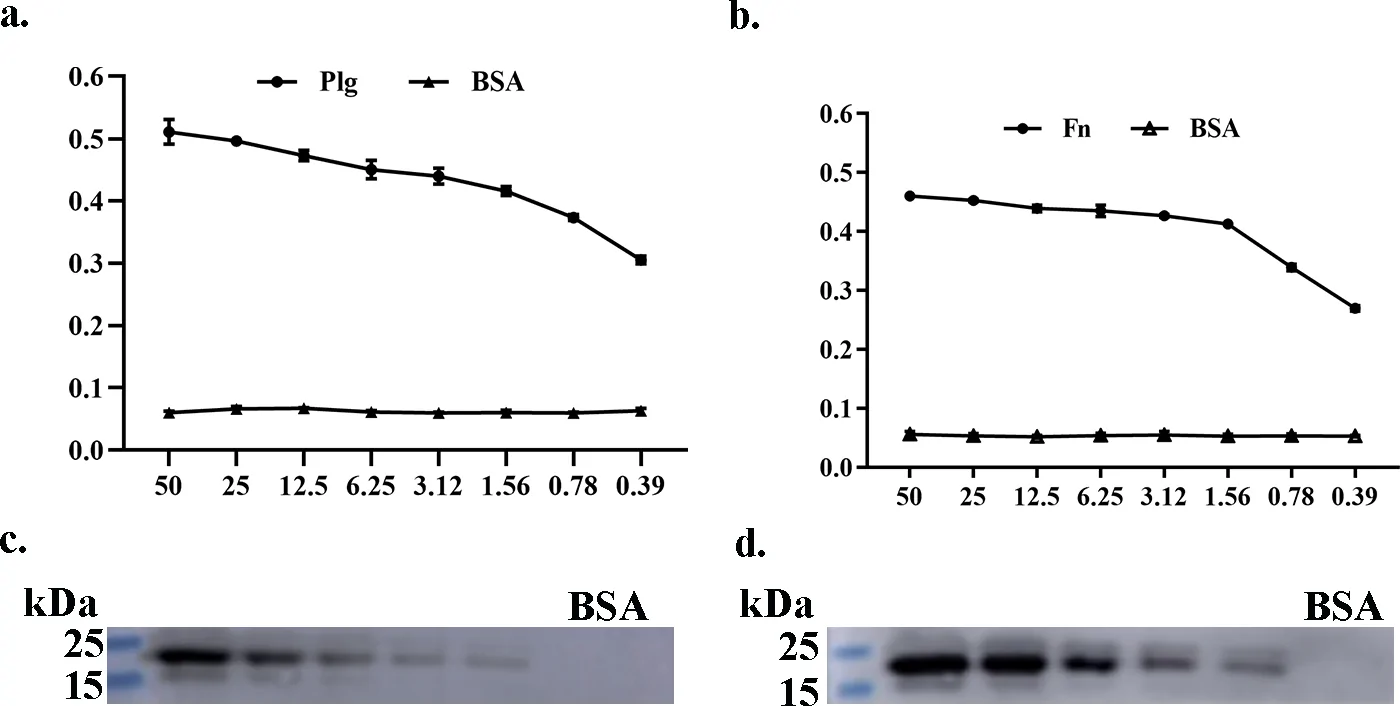
*M. synoviae* SSB interacts with Plg and Fn. (**a**) The binding of Plg to SSB was detected with ELISAs. (**b**) The binding of Fn to SSB was detected with ELISAs. (**c**) The binding of Plg to SSB was detected with Western blotting. (**d**) The binding of Fn to SSB was detected with Western blotting.

Western blot and ELISA analyzes of the binding effect of SSB to Plg and Fn were performed. The results showed that SSB interacts with Plg and Fn in a dose-dependent manner and that the binding dose of Plg and Fn decreased as the amount of SSB decreased, whereas the negative control BSA did not react with SSB (Fig. 3a through d).

### Anti-SSB serum inhibits SSB and *M. synoviae* adhesion to DF-1

**Fig 4:**
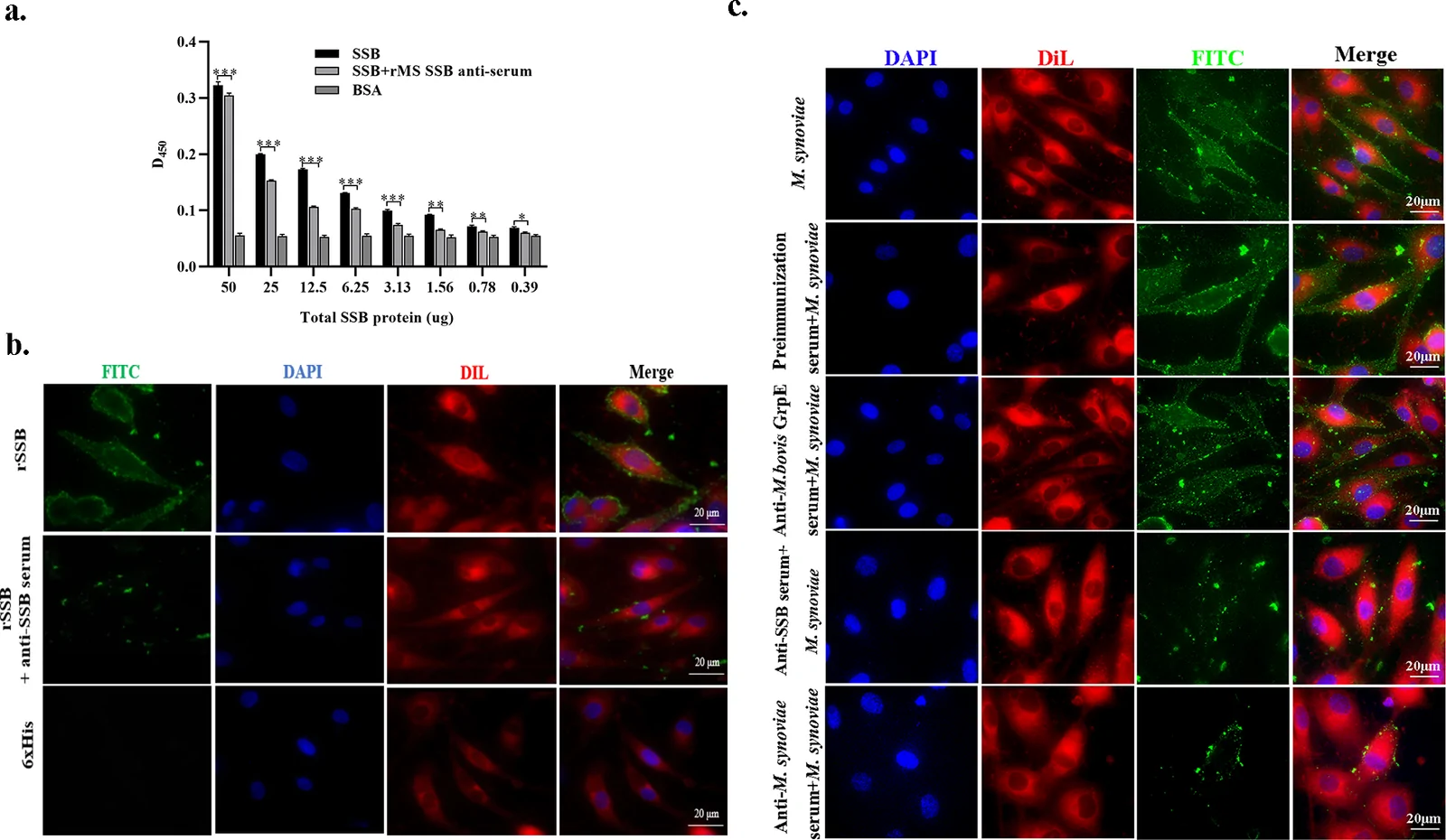
Anti-SSB serum inhibition of SSB and *M. synoviae* adhesion to DF-1 cells. (**a**) ELISA was used to detect SSB binding to DF-1 cell membrane proteins. (**b**) Immunofluorescence detection of the effects of SSB on DF-1 adhesion. The cells were observed under a fluorescence microscope. Red and blue represent cell membrane and nuclei, respectively, and green represents SSB or *M. synoviae*. (**c**) Anti-SSB serum inhibits the adhesion of *M. synoviae* to DF-1 cells.

ELISAs revealed that SSB binds to cell membrane proteins, but anti-SSB serum significantly inhibited the binding of SSB to DF-1 membrane proteins (Fig. 4a). Using the number of *M. synoviae* adherent cell colonies as a 100% adhesion rate, the results showed that the anti-SSB serum inhibited *M. synoviae* adhesion by 33.95% (Table 3). These results indicated that anti-SSB serum could significantly inhibit *M. synoviae* adhesion.

**TABLE 3:** Anti-SSB serum adhesion inhibition test^*b*^

Experimental group	Mean *M. synoviae* colony number (CFU: 1 × 10^3^)	Adherence inhibition rate (%)^*c*^
Anti-SSB serum + *MS*	457 ± 11.59^*a*^	33.95
Anti-*MS* serum *+ MS*	259 ± 13.11^*a*^	62.57
Negative serum	692 ± 17.72^*a*^	/
Colony number blank control	0	/

^*a*^ Results were obtained through three replicate experiments.

^*b*^ *MS*, *Mycoplasmopsis synoviae*.

^*c*^ /, the adherent *M. synoviae* cell colonies of the negative serum and blank groups were used to define the 100% adhesion-rate reference.

To localize the proteins, after incubation of DF-1 cells with SSB, FITC labeling revealed that SSB adhered to the cells. However, compared to the use of anti-GrpE serum (*M. bovis*) and the preimmune serum, the green fluorescence around the cells was significantly reduced after SSB and *M. synoviae* were preincubated with anti-SSB serum before cell incubation (Fig. 4b and c).

### SSB enhances AnxA2 expression and promotes the enrichment of AnxA2 on the cell membrane through colocalization

**Fig 5:**
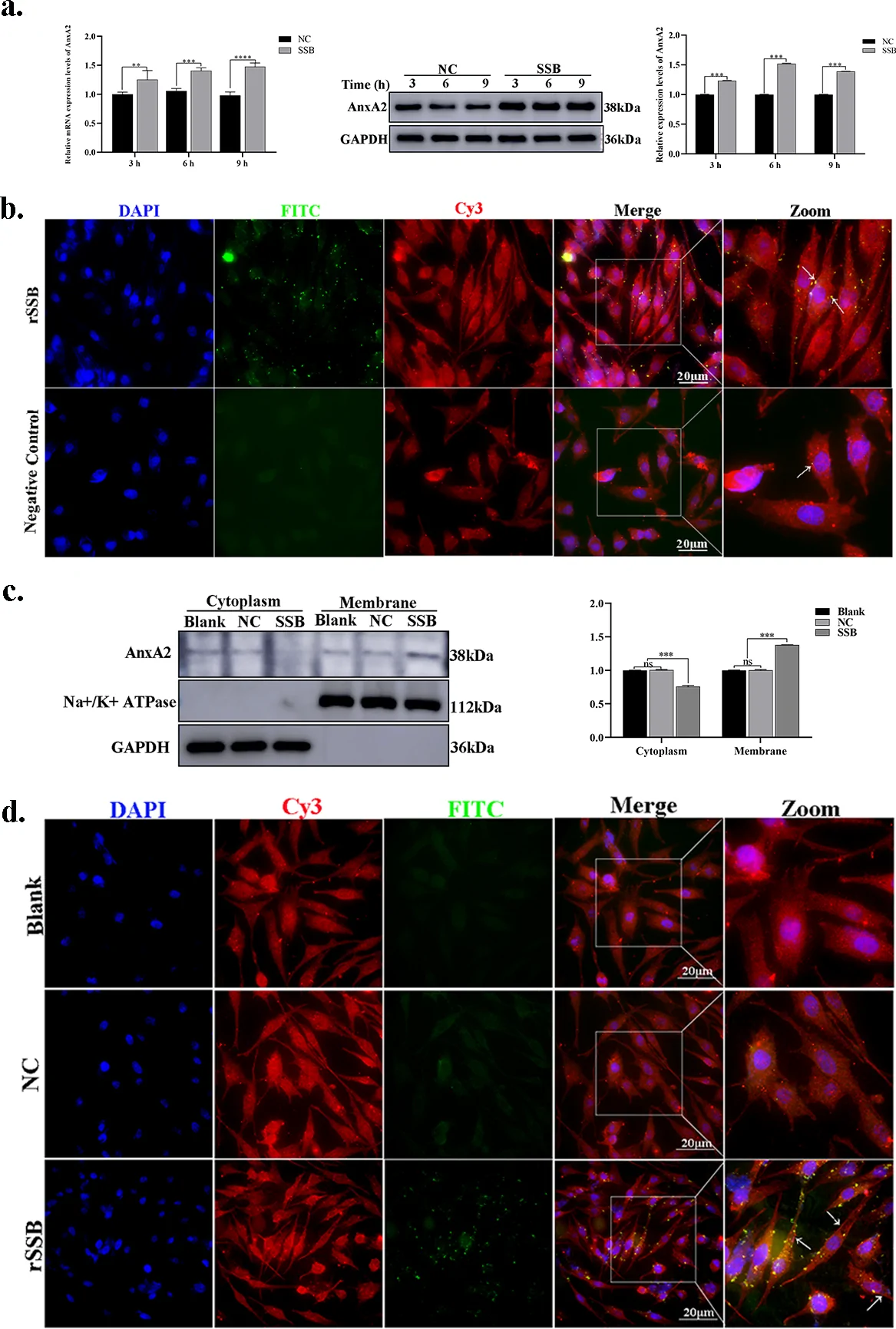
SSB enhances AnxA2 expression and promotes its enrichment on the cell membrane through colocalization. (**a**) Changes in AnxA2 mRNA and protein expression levels were detected by RT‒qPCR and Western blot, and the gray values of the Western blot bands were quantified using ImageJ software. (**b**) Immunofluorescence analysis of *M. synoviae* SSB colocalizes with AnxA2. (**c**) Western blot analysis of SSB-mediated promotion of AnxA2 enrichment at the cell membrane and ImageJ-based quantitative analysis of the gray values of the Western blot bands. (**d**) Immunofluorescence analysis of SSB-mediated promotion of AnxA2 enrichment at the cell membrane. No significant difference, ns; *P* ≥ 0.05; **P < 0.01; ***P < 0.001 and ****P < 0.0001).

Preliminary screening indicated AnxA2 as a potential interacting protein of SSB (relevant data are unpublished). To determine whether SSB regulates host AnxA2, this study further analyzed changes in AnxA2 expression levels and subcellular localization. The RT‒qPCR analysis results revealed that the AnxA2 mRNA expression level increased with increasing SSB duration compared with that in the negative control group, and the difference was statistically significant at 3 h (*P* < 0.01). Furthermore, Western blot analysis revealed that AnxA2 protein expression levels in DF-1 cells gradually increased accordingly (Fig. 5a). These findings suggest that SSB may increase AnxA2 protein expression by increasing AnxA2 transcription.

To examine the colocalization of SSB with AnxA2, immunofluorescence assays were conducted, with empty pET-28a(+) products used as the negative control. The analysis revealed a partial overlap between green fluorescence-labeled SSB and red fluorescence-labeled AnxA2 (Fig. 5b), suggesting that SSB is partially colocalized with AnxA2 in DF-1 cells.

Additionally, treatment with SSB significantly altered the subcellular localization of AnxA2 (*P* < 0.001) (Fig. 5c). Consistent with the Western blot findings, the immunofluorescence data confirmed that, compared with the negative control, SSB facilitates the translocation of the AnxA2 protein from the cytoplasm to the cell membrane, increasing its accumulation at the cell membrane (Fig. 5d). These findings suggest that SSB may influence the adhesion of *M. synoviae* by interacting with AnxA2 and modulating its subcellular localization.

## DISCUSSION

Secreted bacterial proteins from pathogens are mainly adhesins, catabolic enzymes, toxins, and virulence-determining enzymes, which play key roles in bacterial pathogenesis by participating in the adhesion, invasion, proliferation, and inhibition of the host immune response (32). In recent years, important progress has been made in the study of secretory proteomics of various *Mycoplasma* species. For example, *M. bovis* and *M. mycoides* subsp. *mycoides* have 55 and 44 secreted proteins (33), respectively, and *M. hyopneumoniae* and *Mycoplasma flocculare* in swine have 62 and 26 secreted proteins (34), respectively. Among them, the *M. bovis*-secreted proteins MbovP274 and MbovP570 are immunogenic (35). The secreted proteins phosphonate ABC transporter phosphonate-binding protein and BspA family leucine-rich repeat surface protein have been identified as potential molecular markers of *Mycoplasma mycoides* subsp. *mycoides* infection (33). Notably, SSB was not identified among the secreted proteins documented in the above secretome data sets of these mycoplasma species. These findings clearly demonstrate that mycoplasma-secreted proteins exhibit species-specific characteristics in terms of distribution, abundance, and function. Not only do they serve as potential molecular diagnostic markers but they also lay a theoretical foundation for the screening of candidate antigens and further investigation into the pathogenic mechanisms of mycoplasmas.

Distinguishing between actively secreted proteins and intracellular proteins released by bacterial lysis has long been a challenge in mycoplasma secretome research. In 2012, Rebollo Couto et al. successfully cultured *M. synoviae* strain 53 using a modified protein-free Frey medium, concentrated the supernatant using an ultrafiltration system, and then precipitated extracellular proteins via TCA-acetone precipitation. They confirmed, using two-dimensional electrophoresis, that the single-stranded DNA-binding protein is a secreted protein (31). In the present study, we supplemented mycoplasma broth with 10% swine serum to support normal growth. Whole cells were first removed by low-speed centrifugation, and supernatant proteins were then enriched via TFF for mass spectrometry identification. However, during the TFF filtration process, the high-speed circulation of fluid between the feed pump and the membrane module generates significant shear forces, which can easily cause membrane rupture in mycoplasma, leading to the leakage of cytosolic proteins. To minimize protein operation-induced bacterial lysis as much as possible, all procedures in this study were performed in an ice bath, and transmembrane pressure and circulation flow rates were strictly minimized. Additionally, drawing on established quality control protocols for the *M. bovis* secretome (36), colony-forming unit (CFU) counts were used to quantify the number of viable *M. synoviae* before and after centrifugation, thereby assessing the risk of cell membrane rupture induced by mechanical shear forces. The results showed no significant decrease in viable cell counts after completion of the entire pretreatment process. Furthermore, CFU testing of the supernatant after centrifugation demonstrated that by controlling the number of residual live bacteria in the supernatant, the extent of cytoplasmic protein leakage was kept at a low level. Even with the risk of lysis reduced through multiple optimizations, trace amounts of mycoplasma rupture caused by fluid shear cannot be completely eliminated, which is an unavoidable limitation of this method. In addition, the high abundance of proteins in swine serum may interfere with the detection of low-abundance secreted proteins, potentially compromising the accuracy of the experimental results. Despite this challenge, we conducted the first comprehensive characterization of proteins secreted by *M. synoviae* strain WVU1853, successfully identifying 13 proteins. Among these proteins, EF-TU is an immunogenic protein that regulates immune responses in *M. synoviae* (37) and functions as an adhesion factor in *Mycoplasma hyopneumoniae*. EF-TU not only binds to fibronectin in the host’s extracellular matrix, facilitating pathogen colonization, but also interacts with the host’s factor H, thereby evading complement activation and subsequent pathogen elimination (38, 39). Furthermore, the ABC transporter ATP-binding protein of *Mycoplasma bovis* (Mbovp0581) has been identified as an immunogenic secreted protein and is predicted to function as a virulence-associated factor, potentially playing a significant role in the pathogenic mechanisms in this species (40).

In this study, LC‒MS/MS analysis revealed a high coverage rate of polypeptide sequences for SSBs. Subsequent Western blot was employed to confirm that SSB was indeed secreted robustly, thereby reinforcing the reliability of our proteomic analysis. In alignment with the findings of Rebollo Couto et al., our results further substantiate the secretion of SSB in *M. synoviae* strain WVU1853 and provide novel insights into its functional characterization in this pathogen.

Adhesion is crucial for *Mycoplasma* invasion of host cells. The extracellular matrix serves as a barrier against *Mycoplasma*, but plasminogen acts as a receptor for certain *Mycoplasma* adhesins, and its activation leads to matrix degradation, weakening tissue defense and facilitating *Mycoplasma* invasion (41). Fibronectin facilitates the interaction of *M. hyopneumoniae* with the epithelial cell integrin β1, aiding in internalization (42). In recent years, a growing body of research has confirmed that “moonlighting proteins” have been proven to drive bacterial pathogenesis by functioning as surface adhesions or secreted signaling agents. The moonlighting proteins are prevalent among mycoplasmas, as these microbes must make efficient use of their drastically reduced genomes. For example, the typical moonlighting protein of *M. synoviae*, NADH, localizes in both the cytoplasm and the membrane and mediates the adhesion process between the mycoplasma and the host (13). In this study, bioinformatics predictions indicated that SSB lacks the N-terminal signal peptide characteristic of classical secretory proteins, suggesting that the protein is not transported via the classical secretory pathway but is primarily released into the extracellular space through the non-classical secretory pathway. Consistent with subcellular fractionation and immunogold labeling results, SSB was not detected on the membrane of *M. synoviae* WVU1853, ruling out the membrane-anchored form of SSB on the *M. synoviae* surface. Subsequently, we found that moonlighting protein SSBs can specifically bind to the host cell membrane proteins and extracellular matrix proteins Plg and Fn, with binding increasing as SSB levels increase. However, during the protein purification process, we observed two molecular weight bands for the recombinant SSB. In Fig. S4c, the purified product primarily appeared as being 25 kDa, whereas in Fig. 3c and d, only a faint 25 kDa band was observed, with the main product being 22 kDa. We speculate that this may be because the N-terminus of the SSB is a DNA-binding domain that is resistant to proteolytic degradation, whereas the disordered C-terminal region is cleaved by endogenous *E. coli* proteases. Notably, both products retain the complete conserved N-terminal domain and therefore will not interfere with the results of subsequent functional assays. These results confirm that SSB mediates the adhesion of *M. synoviae* to DF-1 cells and suggest that SSB may be closely associated with the internalization and invasion of *Mycoplasmopsis synoviae*.

Due to lack of a cell wall, mycoplasma adhesins can bind to extracellular matrix components or cell surface receptors with the accessory proteins, thereby colonizing the site of interaction (9). This interaction also triggers changes in the host cell’s ultrastructure, induces cytoskeletal reorganizations, and activates various enzymes (43). The molecules that mediate the interaction between mycoplasmas and the extracellular matrix primarily include membrane surface proteins and secreted proteins. In recent years, the role of secreted proteins in the mycoplasma adhesion process has gradually gained attention, and some researchers have proposed a model: mycoplasmas release secreted proteins with adhesive properties that can limit the bacteria’s adhesion to host cells and tissues, thereby promoting the spreading of *Mycoplasma* within the host (44). This study confirms that secreted protein SSB mediates adhesion, and anti-SSB serum significantly inhibits the adhesion of the *M. synoviae* WVU1853 strain. This may be because SSB binding to host cells induces the formation of a microenvironment on the cell surface that is conducive to *M. synoviae* WVU1853 colonization and spreading; when antibodies neutralize SSB, the host cells lose the prerequisites for interaction, and *M. synoviae* adhesion is consequently inhibited.

AnxA2 is a calcium-dependent phospholipid-binding protein that is extensively distributed across the nucleus, cytoplasm, and cell membrane surface (45). It plays crucial roles in cell proliferation, apoptosis, migration, membrane repair, and immunosuppression (46). Previous studies have demonstrated that Fn and tissue plasminogen activator (tPA) facilitate the conversion of Fn into active fibrinolytic enzymes by utilizing the AnxA2 heterotetramer protein complex as a coreceptor, thereby increasing fibrinolysis (46). Furthermore, *Mycoplasma bovis* infection upregulates AnxA2 expression in bovine lung epithelial cells, promoting its translocation from the cytoplasm to the cell membrane (47). Additionally, the N-terminus of the *Mycoplasma hyorhinis* membrane protein P37 interacts with host AnxA2 (48), and the downregulation of host AnxA2 expression has been shown to inhibit *Mycoplasma hyorhinis* infection in human hepatocellular carcinoma (49). In the present study, SSB significantly upregulated AnxA2 expression in DF-1 cells during adhesion, colocalized with AnxA2, and induced AnxA2 enrichment at the cell membrane. The results of this study suggest that the secreted protein SSB may promote *Mycoplasma synoviae* infection by modulating Annexin (AnxA2) expression and localization. Nonetheless, further research is necessary to determine whether AnxA2 enhances *M. synoviae* infection by assisting tPA in converting Plg to plasmin.

According to Falkow’s molecular postulates, to definitively identify a protein as a key virulence factor, gene knockout, gene restoration, and in *vivo* infection model validation must be completed. Due to the limitations of the current experimental conditions, *ssb* knockout and rescue strains have not yet been constructed nor have *in vivo* infection experiments in poultry been conducted. At present, relevant functional results have been obtained only through in *vitro* experiments. The existing evidence can only support functional inferences at the in *vitro* level and does not fully meet all the criteria of the Falkow molecular postulates. In future studies, a series of experiments combining genetic manipulation with *in vivo* infection in poultry can be conducted to further substantiate the virulence role of this protein.

### Conclusions

Overall, this study identified 13 proteins secreted by *Mycoplasma synoviae*. SSB, as a potential virulence factor, is distributed mainly in the cytoplasm and culture supernatant of *M. synoviae*. Additionally, SSB interacts with Plg and Fn, binds to DF-1 cell membrane proteins, and adheres to the cell surface. During adhesion, SSB significantly increases AnxA2 expression levels and promotes its enrichment on the cell membrane surface through colocalization. These findings provide a new theoretical basis for elucidating the biological functions of *M. synoviae*-secreted proteins and their pathogenic mechanisms.

## Supplementary Material

Reviewer comments

## Data Availability

The data sets supporting the conclusions of this article are included within the article and its supplemental materials.

## AFTER EPUB

*[This article was published on 27 August 2026 with an error in Figure 4. The Figure 4 was corrected in the current version, posted on 21 September 2026.]*

## SUPPLEMENTAL MATERIAL

Supplemental figures spectrum.00858-26-s0001.docx Fig. S1 to S4.

Supplemental legends spectrum.00858-26-s0002.docx Legends for Fig. S1 to S4, and Tables S1 and S2.

Table S1 spectrum.00858-26-s0003.xlsx Full peptide identification raw data.

Table S2 spectrum.00858-26-s0004.xlsx MS WVU1853 specific protein identification results.

OPEN PEER REVIEW reviewer-comments.pdf An accounting of the reviewer comments and feedback.

ASM does not own the copyrights to Supplemental Material that may be linked to, or accessed through, an article. The authors have granted ASM a non-exclusive, world-wide license to publish the Supplemental Material files. Please contact the corresponding author directly for reuse.
