# Supplementary material for: A secreted protein of Mycoplasmopsis synoviae, SSB, is a critical adhesion factor that promotes the enrichment of Annexin A2 at the cell membrane
Source: Microbiol Spectr. 2026 Aug 27;14(10):e00858-26. doi: 10.1128/spectrum.00858-26 (PMC13688872; doi:10.1128/spectrum.00858-26)
Supplement: Supplementary file 1 [file reviewer-comments.pdf]

ASM | Microbiology Spectrum

A Secreted Protein of *Mycoplasmopsis synoviae*, SSB, is a Critical Adhesion Factor that Promotes the Enrichment of Annexin A2 at the Cell Membrane

Qing Wang, Yunhai Zhao, Haiyun Ma, xiao he, Yuting Zhang, Xiaoyong Xing, Guomei Quan, Duoduo Si, Zhi Zhang, Shijun Bao, and Xiaochun Wu

Corresponding Author(s): Shijun Bao, Gansu Agricultural University

Review Timeline:

Submission Date:	March 10, 2026
Editorial Decision:	May 15, 2026
Revision Received:	July 14, 2026
Accepted:	August 1, 2026

Editor: John Atack

Reviewer(s): Disclosure of reviewer identity is with reference to reviewer comments included in decision letter(s). The following individuals involved in review of your submission have agreed to reveal their identity: Jiakui L (Reviewer #3)

Transaction Report:

(Note: With the exception of the correction of typographical or spelling errors that could be a source of ambiguity, letters and reports are not edited. The original formatting of letters and referee reports may not be reflected in this compilation.)

DOI: https://doi.org/10.1128/spectrum.00858-26

Re: Spectrum00858-26 (**A Novel Secreted Protein of *Mycoplasma synoviae*, Single-Stranded DNA-Binding Protein, is a Critical Adhesion Factor that Promotes the Enrichment of Annexin A2 at the Cell Membrane**)

Dear Dr. Shijun Bao:

Thank you for the privilege of reviewing your work. Below you will find my comments, instructions from the Spectrum editorial office, and the reviewer comments.

Please pay particular attention to reviewer comments relating to extra experimental controls for the conclusions to be valid

Please return the manuscript within 60 days; if you cannot complete the modification within this time period, please contact me. If you do not wish to modify the manuscript and prefer to submit it to another journal, notify me immediately so that the manuscript may be formally withdrawn from consideration by Spectrum.

Revision Guidelines
To submit your modified manuscript, log into the submission site at https://spectrum.msubmit.net/cgi-bin/main.plex. Go to Author Tasks and click the appropriate manuscript title to begin. The information you entered when you first submitted the paper will be displayed; update this as necessary. Note the following requirements:

• Upload point-by-point responses to the issues raised by the reviewers in a file named "Response to Reviewers," NOT in your cover letter.
• Upload a compare copy of the manuscript (without figures) as a "Marked-Up Manuscript" file.
• Upload a clean .DOC/.DOCX version of the revised manuscript and remove the previous version.
• Each figure must be uploaded as a separate, editable, high-resolution file (TIFF or EPS preferred), and any multipanel figures must be assembled into one file.
• Any supplemental material intended for posting by ASM should be uploaded with their legends separate from the main manuscript. You can combine all supplemental material into one file (preferred) or split it into a maximum of 10 files with all associated legends included.

For complete guidelines on revision requirements, see our Submission and Review Process webpage. Submission of a paper that does not conform to guidelines may delay acceptance of your manuscript.

Data availability: ASM policy requires that data be available to the public upon online posting of the article, so please verify all links to sequence records, if present, and make sure that each number retrieves the full record of the data. If a new accession number is not linked or a link is broken, provide Spectrum production staff with the correct URL for the record. If the accession numbers for new data are not publicly accessible before the expected online posting of the article, publication may be delayed; please contact production staff (Spectrum@asmusa.org) immediately with the expected release date.

Publication Fees: For information on publication fees and which article types are subject to charges, visit our website. If your manuscript is accepted for publication and any fees apply, you will be contacted separately about payment during the production process; please follow the instructions in that e-mail. Arrangements for payment must be made before your article is published.

ASM Membership: Corresponding authors may join or renew ASM membership to obtain discounts on publication fees. Need to upgrade your membership level? Please contact Customer Service at Service@asmusa.org.

The ASM Journals program strives for constant improvement in our submission and publication process. Please tell us how we can improve your experience by taking this quick Author Survey.

Thank you for submitting your paper to Spectrum.

Sincerely,
John Atack
Editor
Microbiology Spectrum

Reviewer #1 (Comments for the Author):

The manuscript by Wang and colleagues presents data on the identification and characterization of Mycoplasma synoviae single-stranded DNA-binding protein (SSB) as a secreted protein that binds to eukaryotic target proteins. The pathogen in question is an important blight to the global poultry industry and its virulence mechanisms are poorly understood. This reviewer identified several scientific questions that require addressing, which are listed below. A number of suggested language revisions

are also included in a separate list.
Scientific Points
1. In the title and in the text, I would also refer to the organism by its new name, Mycoplasmopsis synoviae, at least on first mention.
2. L63 As written, it reads as though MS is the smallest prokaryote. The authors probably meant that "mycoplasmas" are amongst the smallest (with the nanoarchaea).
3. L65 MS also causes tenosynovitis and respiratory lesions
4. L91. The authors use the example of SSBP1 as a pathogenic SSB. They should mention that this is of mitochondrial origin, and include examples of SSN being a virulence factor of certain microbes, as identified using Virulent Pred.
5. L137 It would be informative to add a sentence to explain why overlap PCR was necessary to change the TGA codon
6. L644 Was the protein purified from supernatant or sediment? This should be included L144
7. The subcellular localization studies require controls that are known to be uniquely cytoplasmic and exclusively membrane associated.
8. L218 Ideally sera against a specific irrelevant protein should be included. The anti-MS sera will have a diverse array of IgGs against many antigens-so the comparison with polyclonal serum against SSB (which will have multiple epitopes recognized of one antigen) is not equivalent
9. L269 How do the authors control for cellular lysis with subsequent identification of stable or abundant cytoplasmic proteins?
10. Please mention whether the small SSB only had the regions typical of a standard SSB subch as that from E. coli or B. subtilis (i.e. no extra functional domains are present).
11. L272 As mentioned elsewhere, please give examples of microbial pathogens that use SSB as a virulence factor
12. L284 Please mention the predicted size of the rSSB (with His tag)
13. L288 This seems to contradict the main theme of the ms, that SSB is robustly secreted
14. L266 How do the 13 proteins compare with those that were identified for the other strain in ref 27? Perhaps this could be denoted in the Table 2, together with the extent of coverage with peptides.
15. L302 The authors do not speculate/discuss why SSB specific IgGs reduce adherence of MS. This is important as the authors point o SSB as being either cytoplasmic or fully secreted. Are there predicted bridging molecules involved? Released DNA?
16. L314 Please mention why AnxA2 was suspected as being a candidate for study
17. L314 The expression study requires an irrelevant protein as a control to show that it is SSB specific. Also L321 and L324. PBS is not a suitable control for a polypeptide.
18. The authors should include the term "moonlighting protein" and perhaps raise the possibility that this might be common in mycoplasma since they have reduced protein repertoires
19. L338 and L339. Please specifically include whether SSB does or does not feature in lists of secreted proteins in other mycoplasmas
20. Has SSB ever been reported before as secreted, apart from ref 27?
Language Points
21. SSB has been identified as a secreted protein of M. synoviae in a previous study, so I would not use the word "novel" to describe it.
22. L30 please use the abbreviation SSBs
23. L32 and are potential virulence factors
24. L33 SSB from Escherichia coli, rabbit anti-SSB
25. L37 of chicken DF-1 cells
26. L38 Depending on journal style, "in vitro" and "in vivo" may require italicization
27. L40 promoted Annexin A2 (AnxA2) protein expression
28. L50 are still incomplete. In this study... (I would avoid using "lacking" as there are published studies
29. L53 in the infection process, in vitro. Our results
30. L61 Class does not require italics
31. Depending on journal style, I would introduce spaces ahead of each reference after the final letter of the preceding word
32. L67 infection can lead to coinfection
33. L70 should lay a theoretical
34. L78 because they are intimately involved in a variety of
35. L80 Mycoplasmas should not be italicized when using the term as a trivial name
36. L80 For example, in M. bovis, MbovP280....
37. L88 Single strand DNA-binding proteins (SSBs) are
38. L94 on SSB function in M. synoviae
39. L97 in the cytoplasm and culture
40. L98-99 Please add spaces before parentheses (three instances)
41. L99 Please mention avian source of cells
42. L106 It would be useful to include year of isolation
43. L107 onwards. Depending on journal style, it might be necessary to include City and Country for each company (only on first mention), or city, state and country (if within USA)
44. L109 Escherichia (first mention in the main text)
45. L113 liquid
46. L127 Please include the company and the databases used.
47. L136 cloning

48. L141 After sequence verification of a recombinant clone, the plasmid was transformed
49. L143 How long was induction 3h? O/n?
50. L144 Please include cell lysis and clarification method used
51. L145 and analyzed for purity using a
52. L148 Preparation of poly...
53. L151 sera
54. L153 into rabbits at a
55. The company locations are not required on subsequent mentions
56. L156 was boosted every 7
57. L163 Please define PBS on first mention; PVDF (L190), HRP (L187), BSA (L195), OD (L197), ECL (L192)
58. L173 Please include the commercial source of antibodies, L178 the protease inhibitor used; also L187, also L253/4 (three antisera), L256
59. L181 protein fractions were divided
60. L213 Avoiding light, 3.3...
61. L232 Please include the source of RT, RIPA (L236), Image J (L238), L262 GraphPad
62. L242 Please rephrase "crawled"
63. L246 an Ultra (also L257)
64. L251 Which kit?
65. L260 Please define SEMs
66. L282 of the E. coli
67. L285 transformed with the empty
68. L315 compared with that
69. L319 transcription
70. L333 Secreted bacterial proteins from pathogens are mainly adhesins, catabolic enzymes, toxins and ..
71. L337 These genera can be abbreviated to M.
72. L337 subsp. does not require italicization; also L343 (the species name should not be capitalized)
73. L339 flocculare in swine have
74. L340 Among them, the M. bovis secreted proteins
75. L344 Please rephrase this sentence as the authors intended meaning was not clear to me
76. L347 identified SSB (or is it plural? Were more than one type of SSB identified)? Also match protein/proteins as singular or plural depending on whether SSB or SSBs is used
77. L358 Please include locus tag or other identifier for this ABC ATP cassette as there will be multiple in M. bovis
78. L361 pathogenic mechanisms in this species [31].
79. L361 The sentence that starts "In this study" is disconnected from the previous one. Please modify the transition or use a new paragraph
80. L362 for SSBs.
81. L367 characterization in this pathogen.
82. L369 receptor for certain Mycoplasma
83. L371 spelling of Mycoplasma
84. L372 M. hyopneumoniae with the
85. L380 that Fn and tissue ; L381 Fn
86. L384 Please include a reference for this fact
87. L393 by assisting tPA in converting Plg
88. L399/400 Fn Plg
89. L549 Should Mycoplasma be capitalized (depends how it was published)
90. L574 The restriction enzyme sites are underlined, the mutated site is bolded and italicized
91. L577 supernatant
92. Table 3 colony number Blank control
93. L585 Transmembrane region prediction results for SSB
94. Fig 2 and 3; kDa rather than KDa (also additional file 1
95. L599 Fn to SSB was detected

Reviewer #2 (Comments for the Author):

This study reports a secreted protein of M. synoviae and suggests a role for this protein in pathogenicity. The study is of interest to the readership of the journal and especially to mycoplasmologists and veterinarians. The authors claim that the protein identified is a single stranded binding protein but do not provide functional evidence about the preferred binding of the protein to single stranded DNA over double stranded DNA. They make a reference to the paper Rebello Couto et al. 2008, but this paper does not report the functional confirmation of a secreted SSB in M. synoviae. Therefore, they either rephrase or include additional functionally experiments. In case the protein sequence shows very high similarity to an SSBs for which activity was experimentally confirmed, this should be stated. I suggest the authors have a look at the presence and diversity of the encoding gene in other M. synoviae and Mycoplasma genomes and add these data to the manuscript to get an idea about the

conservation and prevalence of the gene in other strains of the species and in other species. This task can easily be executed and it will add value to the paper.
The methods section needs to be expanded, since it does not provide enough details that allow fellow scientist to replicate experiments in related pathogens. The IACUC number and IACUC committee name for the rabbit immunizations must be provided and the experiment should be described according to ARRIVE guidelines. If the cloning of the antigen in E. coli included codon optimization, which I am only assuming, it should be mentioned. The authors refer to harvesting the cells in the log phase but did not report how the log phase was determined. They have to add how they determined the log phase for their pathogen.
Given the nature of the identified 'secreted' proteins, a critical discussion about pitfalls related to the methods applied should be added. Being devoid of a cell wall means that any little rupture of the cell membrane due to forces exposed by the protocols (mechanical or chemical) would result in leakage of cytoplasmic content into the surrounding media and this should be discussed. Moreover, they should discuss the fact that a signal sequence that is often associated with secreted proteins is not present in the gene sequence encoding their 'secreted' protein. What is the model that enables a secreted protein to act as adhesion in M. synoviae?
The authors claim that the 'Single-Stranded DNA-Binding Protein' investigated in this study is an important virulence factor but do not prove this statement experimentally. This study claims involvement in pathogenicity, which might well, but the study does not at all provide evidence that this factor is an important virulence factor. I advice the authors to study Falkow's molecular postulates and to weigh their data accordingly.

Minor comments
Line 1-3: Change the title and remove the single stranded DNA-binding protein phrase if you cannot prove this function
Line 24-26: I suggest to add the eggshell abnormalities.
Line 63: It is new to me that M. synoviae is the smallest prokaryotic organism, provide a reference if this is really the case.
Line 91: Write ..can increase ...
Line 119: Add how you determined the log phase experimentally.
Line 137-139: If you condon optimized the sequence for expression in E. coli, add this information accordingly.
Line 162: Again, how did you determine the log phase?
Line 169-171: Be specific for example in terms of g-force, amounts of liquids and dilutions of antibodies.
Line 189: Write out fibronectin and plasminogen before you abbreviate
Line 319: Write 'transcription'
Line 361: The mass spectrometry data should be deposited as additional file or uploaded to a database so that it can be accessed by the readership.
Line 599: Change to: The binding of Fn to SSB was detected by ELISAs

I suggest to move Figure 1 to the supplementary material, since it displays only negative in silico data.

Reviewer #3 (Comments for the Author):

Comments for the Author:
Comments for the Author:
The paper titled "A Novel Secreted Protein of Mycoplasma synoviae, Single-Stranded DNA-Binding Protein, is a Critical Adhesion Factor that Promotes the Enrichment of Annexin A2 at the Cell Membrane," following the screening of Mycoplasma synoviae secretory proteins, the authors focused on the gene cloning, protein expression and purification, and antibody preparation of the single-stranded DNA-binding protein (SSB) and subsequently investigated its interaction with host cells. These findings provide a new theoretical perspective for elucidating the pathogenic mechanism of Mycoplasma synoviae and offer potential molecular targets for the prevention of Mycoplasma synoviae infection. The experimental design has clear scientific objectives, and the overall experimental content demonstrates a certain degree of scientific rigor and completeness; however, some details still require refinement. The specific review comments are as follows:

Areas for Improvement and Suggestions for Revision
1. The culture conditions for Mycoplasma synoviae have not yet been clearly defined, including culture temperature and the required gas environment, which still need to be further clarified.
2. The virulence factor predictions have been performed only for single–stranded DNA binding protein among the 13 identified secreted proteins, which fails to highlight the overall value of the screening results. It is recommended that annotations of virulence factor predictions for all secreted proteins in the screening be added to Table 2.
3. Standardize terminology for protein molecular weight units to eliminate inconsistencies and ensure consistent use of standard terms such as "kDa" throughout the manuscript.
4. Number of replicates not specified: Some experiments in the manuscript do not specify the number of replicates (e.g., three independent replicates). The reliability of the experimental results cannot be guaranteed. It is recommended to supplement the manuscript with the number of replicates used for the relevant experiments.
5. Although the manuscript provides RT-qPCR results for Annexin A2 gene expression levels, the experimental section lacks a description of key RT-qPCR parameters. The manuscript should detail the RT-qPCR reaction system and amplification

protocols.
6.Regarding the data results in the manuscript, significant differences are currently indicated only by the symbols "*" or "**" without specifying statistical thresholds. Please supplement the statistical analysis section with an explanation of the P-value criteria and thresholds corresponding to each significance level.
7.The article title is too long. Please simplify it.
8.The language requires further revision.

Title: A Novel Secreted Protein of *Mycoplasma synoviae*, Single-Stranded DNA–Binding Protein, is a Critical Adhesion Factor that Promotes the Enrichment of Annexin A2 at the Cell Membrane

Comments for the Author:

The paper titled "A Novel Secreted Protein of *Mycoplasma synoviae*, Single-Stranded DNA-Binding Protein, is a Critical Adhesion Factor that Promotes the Enrichment of Annexin A2 at the Cell Membrane," following the screening of *Mycoplasma synoviae* secretory proteins, the authors focused on the gene cloning, protein expression and purification, and antibody preparation of the single-stranded DNA-binding protein (SSB) and subsequently investigated its interaction with host cells. These findings provide a new theoretical perspective for elucidating the pathogenic mechanism of *Mycoplasma synoviae* and offer potential molecular targets for the prevention of *Mycoplasma synoviae* infection. The experimental design has clear scientific objectives, and the overall experimental content demonstrates a certain degree of scientific rigor and completeness; however, some details still require refinement. The specific review comments are as follows:

Areas for Improvement and Suggestions for Revision

1. The culture conditions for *Mycoplasma synoviae* have not yet been clearly defined, including culture temperature and the required gas environment, which still need to be further clarified.

2. The virulence factor predictions have been performed only for single–stranded DNA binding protein among the 13 identified secreted proteins, which fails to highlight the overall value of the screening results. It is recommended that annotations of virulence factor predictions for all secreted proteins in the screening be added to Table 2.

3. Standardize terminology for protein molecular weight units to eliminate inconsistencies and ensure consistent use of standard terms such as "kDa" throughout the manuscript.

4. Number of replicates not specified: Some experiments in the manuscript do not

specify the number of replicates (e.g., three independent replicates). The reliability of the experimental results cannot be guaranteed. It is recommended to supplement the manuscript with the number of replicates used for the relevant experiments.

5. Although the manuscript provides RT-qPCR results for Annexin A2 gene expression levels, the experimental section lacks a description of key RT-qPCR parameters. The manuscript should detail the RT-qPCR reaction system and amplification protocols.

6. Regarding the data results in the manuscript, significant differences are currently indicated only by the symbols “*” or “**” without specifying statistical thresholds. Please supplement the statistical analysis section with an explanation of the P-value criteria and thresholds corresponding to each significance level.

7. The article title is too long. Please simplify it.

8. The language requires further revision.

Response to Reviewer's comments

A Secreted Protein of *Mycoplasma synoviae*, Single-Stranded DNA-Binding Protein, is a Critical Adhesion Factor that Promotes the Enrichment of Annexin A2 at the Cell Membrane

Manuscript ID: Spectrum00858-26

Dear Editor and Reviewers:

We appreciate the opportunity to revise our manuscript titled "A Novel Secreted Protein of *Mycoplasma synoviae*, Single-Stranded DNA-Binding Protein, is a Critical Adhesion Factor that Promotes the Enrichment of Annexin A2 at the Cell Membrane" and are grateful for the insightful comments provided for revising and improving our manuscript, as well as the important guiding significance of our work. We have carefully considered all comments and made the necessary revisions on the manuscript. Detailed point-by-point responses to all editorial and reviewers are provided below, preceded by a summary of major revisions. For readability within this response file, editor and reviewer comments are formatted in black font, our responses in blue font, and all revisions in the manuscript in **bold** red font. All page and line numbers cited below correspond to the tracked-changes revised manuscript.

All revisions in the revised manuscript are distinctly highlighted following the rules below:

1. Gray highlight: Deleted text segments.
2. Yellow highlight: All newly added and revised sentences, including reviewer-requested adjustments and self-corrections identified by the authors during proofreading.

We have fully marked all modifications for easy verification and sincerely hope the revised manuscript satisfies the journal's publication requirements.

Response to Reviewer 1……………………………………………………….2-36

Response to Reviewer 2……………………………………………………….37-50

Response to Reviewer 3……………………………………………………….51-55

Reviewer#1

The manuscript by Wang and colleagues presents data on the identification and characterization of *Mycoplasma synoviae* single-stranded DNA-binding protein (SSB) as a secreted protein that binds to eukaryotic target proteins. The pathogen in question is an important blight to the global poultry industry and its virulence mechanisms are poorly understood. This reviewer identified several scientific questions that require addressing, which are listed below. A number of suggested language revisions are also included in a separate list.

Response:

We greatly appreciate the reviewer for spending valuable time evaluating our manuscript and putting forward comprehensive, constructive comments covering both scientific issues and language polishing. We fully recognize the scientific questions raised by the reviewer and will carefully address each point one by one in the revised manuscript. Meanwhile, we have thoroughly revised the manuscript following the provided language editing suggestions to improve its readability and academic standardization.

Scientific Points

Comment 1:

In the title and in the text, I would also refer to the organism by its new name, *Mycoplasmopsis synoviae*, at least on first mention.

Response:

We highly appreciate this important reminder. We have fully modified the new name in our manuscript. The formal scientific name *Mycoplasmopsis synoviae* is used for the first occurrence in both title and text, and unified in subsequent descriptions.

Page **1,** Line [**1**], **the revised objective now reads:**

A Novel Secreted Protein of ***Mycoplasmopsis*** *synoviae*, Single-Stranded DNA-Binding Protein, is a Critical Adhesion Factor that Promotes the Enrichment of Annexin A2 at the cell Membrane.

Comment 2:

L63 As written, it reads as though MS is the smallest prokaryote. The authors probably meant that "mycoplasmas" are amongst the smallest (with the nanoarchaea).

Response:

We thank the reviewer for this careful correction. The original expression incorrectly singled out *M. synoviae* as the smallest prokaryote. We have revised the relevant sentence to avoid inaccurate description as suggested.

Page **4**, Line [**64**], **the revised objective now reads:**

It was first reported as an unknown pathogen in the United States in 1954 and is **one of** the smallest **self-replicating** prokaryotic **organisms** found to date.

Comment 3:

L65 MS also causes tenosynovitis and respiratory lesions

Response:

Thank you for this helpful suggestion. Corresponding information has been added to the manuscript.

Page **4**, Line [**66**], **the revised objective now reads:**

M. synoviae infections in chickens and turkeys result in **tenosynovitis, respiratory lesions,** eggshell apex abnormalities, reduced egg production, and decreased carcass weight in poultry,

Comment 4:

L91. The authors use the example of SSBP1 as a pathogenic SSB. They should mention that this is of mitochondrial origin, and include examples of SSN being a virulence factor of certain microbes, as identified using Virulent Pred.

Response:

Thank you for this valuable comment. We supplemented the text to specify that SSBP1 is mitochondrial-derived and added multiple published studies proving bacterial/

viruses SSB serve as microbial virulence factors. After a thorough literature search, no existing papers have documented SSB predicted as virulence factors by VirulentPred. VirulentPred only provides preliminary in silico screening results that need experimental validation; thus, we relied on functional *in vitro* evidence to support the virulence relevance of SSB instead of unreported VirulentPred prediction data.

Pages **5-6**, Lines [**94, 96-107**], **the revised objective now reads:**

In recent years, it has been shown that **mitochondrial single-strand DNA-binding protein 1** (SSBP1) can increases p53 phosphorylation and its nuclear accumulation through interaction with DNA-dependent protein kinases, which subsequently drives ferroptosis in podocytes. **Concurrently, studies have confirmed that the SSB protein is one of the immunogenic proteins of *Brucella abortus*, capable of eliciting a specific reaction with *Brucella abortus*-positive sera, demonstrating potential for clinical diagnosis. In *Xanthomonas*, the single-stranded DNA-binding protein (SSBx) is a secreted protein dependent on the type-III secretion system (T3SS) that can elicit a hypersensitive response and programmed cell death. Recent research further indicates that a mutation at amino acid position C514 of the herpesvirus single-stranded DNA-binding protein, ICP8, reduces its ability to bind to single-stranded DNA, thereby attenuating the virulence of duck plague virus (DPV) while inducing a potent protective immune response in the host. This provides new targets and important experimental evidence for the development of safe, effective, and non-reverting DPV genetic engineering vaccines.**

Comment 5:

L137 It would be informative to add a sentence to explain why overlap PCR was necessary to change the TGA codon

Response:

Thank you for this constructive comment. We have supplemented a sentence clarifying the necessity of overlap PCR for TGA codon mutation in the revised manuscript.

Page **10**, Lines [**185-187**], **the revised objective now reads:**

The *ssb* sequence contains a TGA codon at 163-165 bp. This codon encodes tryptophan in mycoplasma, whereas in *E. coli* it functions as a stop codon. To achieve site-directed codon mutations within the gene and successfully express SSB,

Comment 6:

L644 Was the protein purified from supernatant or sediment? This should be included L144

Response:

We appreciate this valuable suggestion. The recombinant SSB protein was purified from the soluble after bacterial lysis. We have supplemented this detail at Lines 198-201 of the revised manuscript, and the modified text is shown below:

Pages **10**, Lines [**198-200**], **the revised objective now reads:**

After centrifugation with 10,000×g for 15 min, the supernatant was collected and purified **from the supernatant** using Ni-NTA His-Tag purification resin (MCE, Monmouth Junction, NJ, USA).

Comment 7:

The subcellular localization studies require controls that are known to be uniquely cytoplasmic and exclusively membrane associated.

Response:

We fully agree with your valuable comments. Specific cytoplasmic and membrane protein markers are indeed classic and ideal references for subcellular localization analysis. However, most mycoplasma proteins are moonlighting proteins and are localized to both the cytoplasm and the cell membrane. To accurately and comprehensively characterize the subcellular distribution of SSB, this study combined two methods: Western blotting for detecting the separate cytoplasmic and membrane protein fractions and immunoelectron microscopy imaging. The direct visual evidence provided by immunoelectron microscopy compensates for the limitation of lacking canonical cytoplasmic and membrane marker proteins as internal controls.

Comment 8:

L218 Ideally sera against a specific irrelevant protein should be included. The anti-MS sera will have a diverse array of IgGs against many antigens-so the comparison with polyclonal serum against SSB (which will have multiple epitopes recognized of one antigen) is not equivalent

Response:

We fully agree with this comment that whole anti-*M. synoviae* polyclonal serum contains abundant IgGs targeting dozens of mycoplasma antigens, which cannot serve as a matched negative control for the anti-SSB monospecific polyclonal antibody.

As you rightly pointed out, most mycoplasma proteins act as moonlighting proteins that localize in both the cytoplasm and cell membrane and participate in host adhesion. For this reason, it is extremely difficult to identify truly irrelevant endogenous proteins from *M. synoviae*, and we currently lack immune sera targeting unrelated *M. synoviae* proteins as ideal controls.

To reduce the bias caused by inconsistent antibody profiles between multi-antigen anti-*M. synoviae* whole-cell sera and single-antigen anti-SSB polyclonal sera, we set up two complementary negative controls: pre-immune serum and anti-*Mycoplasma bovis* GrpE serum prepared with the exact same immunization procedure as the anti-serum. Sequence alignment showed only 23.34% amino acid identity between *M. bovis* Grpe and *M. synoviae* Grpe. Western blot verification confirmed that this anti-Grpe serum has no specific reactivity against *M. synoviae* (Supplemental material FIG S2).

We recognize that heterologous anti-GrpE serum cannot fully substitute sera against an irrelevant *M. synoviae* protein. Nevertheless, this dual-control system represents the most feasible alternative under our current experimental conditions to standardize the comparison between single-antigen polyclonal sera and whole-cell antisera. We have now rewritten the relevant sections on methods and results in the revised manuscript.

Page **15**, Lines [**295-297**], **the revised objective now reads:**

Anti-*M. bovis* GrpE (Western blotting confirmed no cross-reactivity of this

antibody against with *M. synoviae*, refer to Supplemental Figures FIG S2), Preimmune serum, anti-SSB and anti-*M. synoviae* sera were diluted (1:50), preincubated with *M. synoviae* at 37 °C for 1 hour.

Page **22**, Lines [**430-431**], **the revised objective now reads:**

However, **compared to the use of anti-GrpE serum (*M. bovis*) and the preimmune serum**, the green fluorescence around the cells was significantly reduced after SSB and *M. synoviae* were preincubated with anti-SSB serum before cell incubation (Fig. 4b and c).

Comment 9:

L269 How do the authors control for cellular lysis with subsequent identification of stable or abundant cytoplasmic proteins?

Response:

We thank the reviewer for this important technical question regarding the discrimination of genuine secreted proteins from cytoplasmic contaminants released by mycoplasma lysis. We have established a multi-layered control strategy to assess and minimize mechanical rupture od wall-less *M. synoviae*, and we have supplemented the full description of these controls and relevant limitations in the revised Discussion section.

Pages **24-26**, Lines [**479-503**], **the revised objective now reads:**

Distinguishing between actively secreted proteins and intracellular proteins released by bacterial lysis has long been a challenge in mycoplasma secretome research. In 2012, Rebollo Couto et al. successfully cultured *M. synoviae* strain 53 using a modified protein-free Frey medium, **concentrated the supernatant using an ultrafiltration system, and then precipitated extracellular proteins via TCA-acetone precipitation. They confirmed, using two-dimensional (2-DE) electrophoresis, that the** single-stranded DNA-binding protein is a secreted protein. In the present study, we supplemented mycoplasma broth with 10% swine serum to support normal growth. **Whole cells were first removed by low-speed centrifugation,**

and supernatant proteins were then enriched via TFF for mass spectrometry identification. However, during the TFF filtration process, the high-speed circulation of fluid between the feed pump and the membrane module generates significant shear forces, which can easily cause membrane rupture in mycoplasma, leading to the leakage of cytosolic proteins. To minimize protein operation-induced bacterial lysis as much as possible, all procedures in this study were performed in an ice bath, and transmembrane pressure and circulation flow rates were strictly minimized. Additionally, drawing on established quality control protocols for the *M. bovis* secretome, Colony-Forming Unit (CFU) counts were used to quantify the number of viable *M. synoviae* before and after centrifugation, thereby assessing the risk of cell membrane rupture induced by mechanical shear forces. The results showed no significant decrease in viable cell counts after completion of the entire pretreatment process. Furthermore, CFU testing of the supernatant after centrifugation demonstrated that by controlling the number of residual live bacteria in the supernatant, the extent of cytoplasmic protein leakage was kept at a low level. Even with the risk of lysis reduced through multiple optimizations, trace amounts of mycoplasma rupture caused by fluid shear cannot be completely eliminated, which is an unavoidable limitation of this method.

Comment 10:

Please mention whether the small SSB only had the regions typical of a standard SSB subch as that from E. coli or B. subtilis (i.e. no extra functional domains are present).

Response:

As review of the literature reveals that SSB recognize and bind to DNA via a conserved oligonucleotide/oligosaccharide-binding fold (OB-fold). This core functional domain contains approximately 100 amino acids and serves as the fundamental structural unit for binding single-stranded DNA and performing biological functions.

The *E. coli* SSB protein consists of 178 amino acids. The N-terminal sequence (residues 1-115) forms a typical OB-fold domain, the C-terminal region (residues 116-178) can

be divided into two subdomains, with residues 116-170 constituting an intrinsically disordered linker (IDL) region responsible for mediating protein-protein interactions; while residues 171-178 form the acidic C-terminal region, which maintains the stability of SSB tetrameric structure. The Bacillus subtilis SSB shares strong sequence similarity with the DNA-binding N-terminal domain and protein-binding C-terminus of SSB $_{Eco}$. Conserved domain analysis via NCBI CDD revealed that the *M. synoviae* SSB contains only the canonical SSB domain. The SSB consists of a core SSB domain (residues 4-108) and an overlapping extended SSB homology domain (residues 55-165), both corresponding to the conserved core oligonucleotide/oligosaccharide-binding fold (OB-fold) region, followed by a short C-terminal segment, consistent with the domain architecture of canonical SSB from *E. coli* and *Bacillus subtilis*. No additional functional domains were identified beyond the conserved C-terminal tail.

Page **20**, Lines [**389-395**], **the revised objective now reads:**

Conserved domain analysis via NCBI CDD revealed that the *M. synoviae* SSB contains only the canonical SSB domain. The SSB consists of a core SSB domain (residues 4-108) and an overlapping extended SSB homology domain (residues 55-165) (Fig 1c), both corresponding to the conserved core oligonucleotide/oligosaccharide-binding fold (OB-fold) region, followed by a short C-terminal segment, consistent with the domain architecture of canonical SSB from *E. coli* and *Bacillus subtilis.* No additional functional domains were identified beyond the conserved C-terminal tail.

Comment 11:

L272 As mentioned elsewhere, please give examples of microbial pathogens that use SSB as a virulence factor

Response:

We greatly appreciate the reviewer's repeated reminder to supplement examples of SSB as a virulence factor, and we fully recognize the insufficient supporting evidence in the initial manuscript. We have comprehensively revised the introduction section **(Pages 5-**

6, lines 96-107) and added multiple verifiable, peer-reviewed references to solidify this ratio.

Comment 12:

L284 Please mention the predicted size of the rSSB (with His tag)

Response:

According to the Uniprot database, the theoretical molecular weight of native SSB is 21.2 kDa. After fusing with 6 × His tag, the predicted molecular weight of the recombinant rSSB is approximately 25 kDa. A specific band at 25 kDa was detected in whole cell lysates, supernatants, and precipitates, consistent with the theoretical molecular weight of the recombinant protein.

Page **21**, Lines [**401-403**], **the revised objective now reads:**

According to the UniProt database, the theoretical molecular weight (MW) of native SSB is 21.2 kDa, After fusing with 6 × His tag, the predicted MW of the recombinant SSB is approximately 25 kDa.

Comment 13:

L288 This seems to contradict the main theme of the ms, that SSB is robustly secreted

Response:

We thank the reviewer for this careful point and clarify that there is no contradiction with the secretion characteristic of native SSB in *M. synoviae* WVU1853.

The statement that SSB localizes exclusively in the cytoplasm only describes the subcellular location of SSB retained inside fixed intact *M. synoviae* cells captured by immunolabeling. This observation reflects the intracellular pool of SSB prior to secretion, rather than indicating that SSB cannot be released extracellularly. The secretome analysis using culture supernatant separately confirms that a fraction of SSB is successfully secreted outside mycoplasma cells, so there is no inherent contradiction between the two databases.

Comment 14:

L266 How do the 13 proteins compare with those that were identified for the other strain in ref 27? Perhaps this could be denoted in the Table 2, together with the extent of coverage with peptides.

Response:

As suggested, we have summarized the comparison between our 13 candidate proteins and those from the other strain in Ref. 31 in Table 2. The peptide coverage of each protein is presented, and proteins absent in the previous dataset are labeled "Not identified".

Page **19**, Lines [**362-364**], **the revised objective now reads:**

We further compared these 13 candidate proteins with homologs reported in another strain and the detailed information including sequence coverage is listed in Table 2.

Pages **40-42**, Lines [**825-828**], **the revised objective now reads:**

TABLE 2 Secreted proteins localized to the supernatants

Accession Number	Protein names	Sequence Coverage	Homologous protein in Ref. 31
AKB11101.1	deoxyribonuclease	**15**	**Not identified**
AKB11382.1	Elongation factor Tu	**11**	**99.75%**
AKB11096.1	VY93_01090 rRNA methyltransferase	**10**	**Not identified**
AKB10884.1	ABC transporter substrate-binding	**9**	**Not identified**

	protein		
AKB11260.1	single-stranded DNA-binding protein	**8**	**100%**
AKB11161.1	DNA helicase	**8**	**Not identified**
AKB11174.1	histidyl-tRNA synthetase	**7**	**Not identified**
AKB10936.1	hypothetical protein VY93_00850	**6**	**Not identified**
AKB10998.1	lipoprotein	**5**	**Not identified**
AKB10971.1	peptide ABC transporter ATP-binding protein11	**5**	**Not identified**
AKB10972.1	hypothetical protein VY93_01090	**2**	**Not identified**
AKB11015.1	lipoprotein	**2**	**Not identified**
AKB11330.1	ABC transporter permease	**1**	**Not identified**

Accession number of proteins and protein names are derived from the NCBI database.

Not identified: it means that this protein was not detected in the proteomic dataset of the other strain reported in Reference 31.

Comment 15:

L302 The authors do not speculate/discuss why SSB specific IgGs reduce adherence of MS. This is important as the authors point o SSB as being either cytoplasmic or fully secreted. Are there predicted bridging molecules involved? Released DNA?

Response:

Thank you much for your valuable comment. We have supplemented the discussion on the potential mechanism in the revised manuscript.

Pages **28-29**, Lines [**555-569**], **the revised objective now reads:**

Due to lack of a cell wall, mycoplasma adhesins can bind to extracellular matrix components or cell surface receptors with the accessory proteins, thereby colonizing the site of interaction. This interaction also triggers changes in the host cell's ultrastructure, induces cytoskeletal reorganizations, and activates various enzyme. The molecules that mediate the interaction between mycoplasmas and the extracellular matrix primarily include membrane surface proteins and secreted proteins. In recent years, the role of secreted proteins in the mycoplasma adhesion process has gradually attention, and some researchers have proposed a model: mycoplasmas release secreted proteins with adhesive properties that can limit the bacteria's adhesion to host cells and tissues, thereby promoting the spreading of *Mycoplasma* within the host. This study confirms that secreted protein SSB mediates adhesions and anti-SSB serum significantly inhibits the adhesion of *M. synoviae* WVU1853 strain. This may be because SSB binding to host cells induces the formation of a microenvironment on the cell surface that is conducive to *M. synoviae* WVU1853 colonization and spreading; when antibodies neutralize SSB, the host cells lose the prerequisites for interaction, and *M. synoviae* adhesion is consequently inhibited.

Comment 16:

L314 Please mention why AnxA2 was suspected as being a candidate for study

Response:

We appreciate this valuable suggestion. We have supplemented relevant explanations in the manuscript to elaborate the rationale for selecting AnxA2 as a candidate protein.

Page **22**, Lines [**437-439**], **the revised objective now reads:**

Preliminary screening indicated AnxA2 as a potential interacting protein of SSB (relevant data are unpublished). To determine whether SSB regulates host AnxA2, this study further analyzed changes in AnxA2 expression levels and subcellular localization.

Comment 17:

L314 The expression study requires an irrelevant protein as a control to show that it is SSB specific. Also L321 and L324. PBS is not a suitable control for a polypeptide.

Response:

We thank the reviewer for this critical suggestion. To confirm the specificity of SSB function, an irrelevant protein control ideal as point out. However, such irrelevant protein is not available in our lab at present. Therefore, we used parallel-purified protein from empty pET-28a (+) vector ad the negative control to exclude non-specific effects derived from the expression system and purification.

Page **23**, Line [**441**], **the revised objective now reads:**

The RT–qPCR analysis results revealed that the AnxA2 mRNA expression level increased with increasing SSB duration compared with that in the **negative** control group,

Page **23**, Line [**447**], **the revised objective now reads:**

To examine the colocalization of SSB with AnxA2, immunofluorescence assays were conducted, with **empty pET-28a products** used as the negative control.

Page **23**, Line [**453**], **the revised objective now reads:**

Consistent with the Western blot findings, the immunofluorescence data confirmed that, compared with the **negative** control,

Comment 18:

The authors should include the term "moonlighting protein" and perhaps raise the possibility that this might be common in mycoplasma since they have reduced protein repertoires

Response:

We have supplemented the term “moonlighting protein” in the revised manuscript. Considering mycoplasmas possess highly reduced protein repertoires, we also discussed the possibility that moonlighting functions may be prevalent among mycoplasma secreted proteins to compensate for their limited coding capacity.

Page **27**, Lines [**529-535**], **the revised objective now reads:**

In recent years, a growing body of research has confirmed that “moonlighting proteins” have been proven to drive bacterial pathogenesis by functioning as surface adhesions or secreted signaling agents. The moonlighting proteins are prevalent among mycoplasmas, as these microbes must make efficient use of their drastically reduced genomes. For example, the typical moonlighting protein of *M. synoviae*, NADH, localises in both the cytoplasm and the membrane and mediates the adhesion process between the mycoplasma and the host.

Comment 19:

L338 and L339. Please specifically include whether SSB does or does not feature in lists of secreted proteins in other mycoplasmas

Response:

We appreciate this valuable comment. As requested, we added the above supplementary sentence to clarify that SSB is absent from the secreted protein lists of multiple other mycoplasma species.

Page **24**, Lines [**471-473**], **the revised objective now reads:**

Notably, SSB was not identified among the secreted proteins documented in the above secretome datasets of these mycoplasma species.

Comment 20:

Has SSB ever been reported before as secreted, apart from ref 27?

Response:

We sincerely appreciate this valuable comment, and we have supplemented comprehensive comparative evidence to explicitly describe the absence of SSB in curated secreted protein inventories of different mycoplasma species in the revised manuscript. In the meantime, we fully supplemented cross-species comparative evidence in the revised manuscript to clarify this point and further introduced the contrasting phenotype in *Xanthomonas* as supportive literature of SSB secretion across bacteria.

Language Points

We sincerely appreciate the reviewer's careful reading and numerous suggestions on English expression. We have thoroughly revised the language throughout the manuscript to improve readability and accuracy, and all linguistic revisions have been marked in the revised version.

Comment 21:

SSB has been identified as a secreted protein of *M. synoviae* in a previous study, so I would not use the word "novel" to describe it.

Page **1**, Line [**1**], **the revised objective now reads:**

A Secreted Protein of *Mycoplasmopsis synoviae*, Single-Stranded DNA-Binding Protein, is a Critical Adhesion Factor that Promotes the Enrichment of Annexin A2 at the Cell Membrane

Comment 22:

L30 please use the abbreviation SSBs

Page **2**, Lines [**30-31**], **the revised objective now reads:**

Bioinformatic analysis of **SSBs** revealed that they lack a transmembrane domain and the characteristic features of a cleavable signal peptide and are virulence factors.

Comment 23:

L32 and are potential virulence factors

Page **2**, Line [**32**], **the revised objective now reads:**

Bioinformatic analysis of single-stranded DNA-binding proteins revealed that they lack a transmembrane domain and the characteristic features of a cleavable signal peptide and are **potential** virulence factors.

Comment 24:

L33 SSB from Escherichia coli, rabbit anti-SSB

Page **2**, Lines [**33-34**], **the revised objective now reads:**

After induced expression and purification of SSB **from *Escherichia coli***, **rabbit** anti-SSB serum was prepared.

Comment 25:

L37 of chicken DF-1 cells

Page **2**, Line [**38**], **the revised objective now reads:**

ELISA and indirect immunofluorescence analyses revealed that SSB binds to membrane proteins and adheres to the surface of **chicken** DF-1 cells and that rabbit anti-SSB serum significantly inhibits *M. synoviae* adhesion to DF-1 cells.

Comment 26:

L38 Depending on journal style, "in vitro" and "in vivo" may require italicization

Page **2**, Line [**39**], **the revised objective now reads:**

In vitro experiments confirmed that SSB could interact directly with plasminogen and fibronectin in a dose-dependent manner.

Comment 27:

L40 promoted Annexin A2 (AnxA2) protein expression

Page **3**, Line [**41**], **the revised objective now reads:**

In addition, our study revealed that SSB significantly promoted Annexin A2 **(AnxA2)** protein expression in DF-1 cells during adhesion, significantly colocalized with AnxA2, and promoted its enrichment at the membrane.

Comment 28:

L50 are still incomplete. In this study... (I would avoid using "lacking" as there are published studies

Page **3**, Line [**52**], **the revised objective now reads:**

However, studies on secreted proteins of *M. synoviae* are still **incomplete**.

Comment 29:

L53 in the infection process, in vitro. Our results

Page **3**, Lines [**54-55**], **the revised objective now reads:**

In our study, we systematically screened and identified the secreted proteins of *M. synoviae* strain WVU1853 for the first time and conducted an in-depth investigation of the role of SSB, one of the secreted proteins, in the infection process**, *in vitro***.

Comment 30:

L61 Class does not require italics

Page **4**, Line [**62**], t**he revised objective now reads:**

Mycoplasma synoviae (*M. synoviae*, *MS*) belongs to the **Class** *Mollicutes* and lacks a cell wall.

Comment 31:

Depending on journal style, I would introduce spaces ahead of each reference after the

final letter of the preceding word

Response:

Thank you for the careful reminder. I have added required spaces before each reference citation in accordance with the journal formatting style in the revised manuscript.

Comment 32:

L67 infection can lead to coinfection

Page **4**, Line [**69**], **the revised objective now reads:**

Notably, immunosuppression after *M. synoviae* infection **can lead** to coinfection with other avian pathogens, such as Newcastle disease virus, infectious bronchitis virus, and avian reovirus, which can significantly aggravate the disease and result in more serious economic losses.

Comment 33:

L70 should lay a theoretical

Page **4**, Line [**72**], **the revised objective now reads:**

Therefore, research on *M. synoviae* **should** lay a theoretical foundation for a deeper understanding of the pathogenesis of *M. synoviae* and the establishment of effective diagnostic methods as well as preventive and control measures.

Comment 34:

L78 because they are intimately involved in a variety of

Pages **4-5**, Lines [**80-81**], **the revised objective now reads:**

In recent years, secreted proteins have gained attention because they are **intimately involved in** a variety of functions, such as nutrient uptake, adhesion and invasion.

Comment 35:

L80 Mycoplasmas should not be italicized when using the term as a trivial name

Response:

Thank you for your valuable suggestion. We have revised this section and carefully checked the entire manuscript accordingly. We strictly distinguished between Latin taxonomic names and common trivial nouns.

Page **5**, Line [**82**], **the revised objective now reads:**

The proteins secreted by several **mycoplasmas** play important roles in pathogenicity.

Comment 36:

L80 For example, in *M. bovis*, MbovP280....

Page **5**, Line [**83**], **the revised objective now reads:**

For example, **in *M. bovis*,** MbovP280 induces apoptosis via its coiled-coil domain and the cellular ligand CRYAB.

Comment 37:

L88 Single strand DNA-binding proteins (SSBs) are

Page **5**, Line [**91**], **the revised objective now reads:**

Single-Strand DNA-binding proteins (**SSBs**) are indispensable in DNA metabolism and can specifically bind to single-strand DNA regions to regulate important processes such as DNA replication, recombination, and repair.

Comment 38:

L94 on SSB function in *M. synoviae*

Page **6**, Line [**108**], **the revised objective now reads:**

However, most SSB-related studies have focused on eukaryotes, and there are no reports on SSB **function in** *M. synoviae*.

Comment 39:

L97 in the cytoplasm and culture

Page **6**, Line [**111**], **the revised objective now reads:**

Among them, the SSB protein, as a potential virulence factor, is distributed mainly in

the cytoplasm and culture supernatants and interacts with plasminogen (Plg) and fibronectin (Fn).

Comment 40:

L98-99 Please add spaces before parentheses (three instances)

Page **6**, Lines [**112-113**], **the revised objective now reads:**

Among them, the SSB protein, as a potential virulence factor, is distributed mainly in cytoplasmic and culture supernatants and interacts with plasminogen **(Plg)** and fibronectin **(Fn)**, significantly adhering to DF-1 cells and promoting Annexin A2 **(AnxA2)** enrichment at the cell membrane by colocalizing with AnxA2.

Comment 41:

L99 Please mention avian source of cells

Page **6**, Line [**113**], **the revised objective now reads:**

Among them, the SSB protein, as a potential virulence factor, is distributed mainly in cytoplasmic and culture supernatants and interacts with plasminogen (Plg) and fibronectin (Fn), significantly adhering to **chicken** DF-1 cells and promoting Annexin A2 (AnxA2).

Comment 42:

L106 It would be useful to include year of isolation

Page 6, Line [**120**], **the revised objective now reads:**

M. synoviae strain WVU1853 was **isolated from chicken joints in the United States in 1957.**

Comment 43:

L107 onwards. Depending on journal style, it might be necessary to include City and Country for each company (only on first mention), or city, state and country (if within USA)

Response:

We have supplemented the city and country information for each reagent supplier at the first occurrence, and added state details for manufacturers located in the United States, complying with the journal formatting requirements.

Comment 44:

L109 Escherichia (first mention in the main text)

Page **7**, Line [**126**], **the revised objective now reads:**

The ***Escherichia*** *coli* **(*E. coli*)** strain BL21 (DE3) was purchased from TransGen Biotech Co., Ltd (Beijing, China).

Comment 45:

L113 liquid

Page **7**, Line [**130**], **the revised objective now reads:**

Chicken embryo fibroblast (DF-1) cells were obtained from Procell (China) cultured in Dulbecco's modified Eagle's medium (GIBCO, USA) supplemented with 10% foetal bovine serum (Biological industries, Shanghai, China) and 1% penicillin-streptomycin **liquid** (Solarbio).

Comment 46:

L127 Please include the company and the databases used.

Page **8**, Lines [**151-154**]), **the revised objective now reads:**

All related bioinformatics analyses were performed using a database from National Center for Biotechnology Information (NCBI, NLM, NIH, Bethesda, MD, USA, accession No. CP011096.1) and commercial services provided by Shanghai OE Biotech Co., Ltd. (Shanghai, China).

Comment 47:

L136 cloning

Page **10**, Line [**184**], **the revised objective now reads:**

SSB **cloning,** expression and purification

Comment 48:

L141 After sequence verification of a recombinant clone, the plasmid was transformed

Page **10**, Lines [**192-194**], **the revised objective now reads:**

After sequence verification of a recombinant clone, the plasmid was transformed into *E. coli* BL21 (DE3),

Comment 49:

L143 How long was induction 3h? O/n?

Page **10**, Lines [**195-196**], **the revised objective now reads:**

, and protein expression was induced using the addition of 1 mM isopropyl β-D-thiogalactoside (IPTG, Solarbio) at 16 ℃ **for 10 hours.**

Comment 50:

L144 Please include cell lysis and clarification method used

Pages **10**, Lines [**196-200**], **the revised objective now reads:**

Cells were harvested by centrifugation at 8,000×g for 10 min and washed three times with pre-cooled PBS. The precipitates were resuspended in 10 mL of binding buffer, followed by ultrasonication for 30 min under the 200 W power. After centrifugation with 10,000×g for 15 min, the supernatant was collected and purified **from the supernatant** using Ni-NTA His-Tag purification resin (MCE, Monmouth Junction, NJ, USA).

Comment 51:

L145 and analyzed for purity using a

Page **11**, Line [**201**], **the revised objective now reads:**

The purified target protein was quantified and **analyzed for purity** using a BCA protein

assay kit (Beyotime, Shanghai, China) and SDS–PAGE, respectively.

Comment 52:

L148 Preparation of poly...

Page **11**, Line [**204**], **the revised objective now reads:**

Preparation of polyclonal anti-SSB serum

Comment 53:

L151 sera

Page **11**, Line [**207**], **the revised objective now reads:**

The specific steps were as follows: Prior to immunization, one millilitre of blood was collected from the auricular vein of the ears of New Zealand white rabbits as negative control **sera**.

Comment 54:

L153 into rabbits at a

Page **11**, Line [**210**], **the revised objective now reads:**

The purified protein was then mixed with an equal volume of Freund's complete adjuvant (Sigma-USA), fully emulsified, and injected subcutaneously at multiple points **into rabbits at** a dose of 800 μg.

Comment 55:

The company locations are not required on subsequent mentions

Response:

We have adjusted accordingly. The company locations are only retained for the first citation and omitted in subsequent appearances.

Comment 56:

L156 was boosted every 7

Page **11**, Line [**213**], **the revised objective now reads:**

Subsequently, immunization was **boosted** every 7 days using the same dose and method as noted for the second immunization.

Comment 57:

L163 Please define PBS on first mention; PVDF (L190), HRP (L187), BSA (L195), OD (L197), ECL (L192)

Page **13**, Line [**255**], **the revised objective now reads:**

SSB was transferred to **polyvinylidene fluoride membrane** (PVDF) in varying amounts (10 µg to 0.625 µg), and Plg or Fn was bound at 37 °C for 2 hours.

Page **13**, Line [**259**], **the revised objective now reads:**

Anti-SSB serum was used as the primary antibody, and goat anti-rabbit **horseradish peroxidase** (HRP)/IgG was used as the secondary antibody.

Page **14**, Line [**264**], **the revised objective now reads:**

After the samples were blocked with 5% nonfat milk, SSB (50 µg to 0.39 µg) was added to each well, with **bovine serum albumin** (BSA) used as a negative control.

Page **14**, Line [**267**], **the revised objective now reads:**

SSB binding to Plg and Fn was assessed by using **optical density 450** (OD_{450}) absorbance measurements.

Page **14**, Line [**261**], **the revised objective now reads:**

Enhanced chemiluminescence luminescent solution (ECL) (Oriscience, Chengdu, China) was used to visualize the protein bands.

Comment 58:

L173 Please include the commercial source of antibodies, L178 the protease inhibitor used; also L187, also L253/4 (three antisera), L256

Page **12**, Line [**238**], **the revised objective now reads:**

; preimmunization serum as a negative control and anti-*M. synoviae* serum as a positive control), incubated with goat anti-rabbit IgG/FITC antibodies **(Bioss, Beijing, China)** as secondary antibodies,

Page **13**, Line [**242**], **the revised objective now reads:**

Then, 1 mM protease inhibitor **(Solarbio)** and 4% Triton X-114 were added, and the mixture was incubated at 4 °C for 30 min.

Page **13**, Line [**251**], **the revised objective now reads:**

Anti-SSB serum was used as the primary antibody, and goat anti-rabbit HRP/IgG **(Bioss)** was used as the secondary antibody.

Page **18**, Lines [**344-345**], **the revised objective now reads:**

GAPDH **(Abmart, Shanghai, China)** and Na^+/K^+-ATPase **(Selleck Chemicals, Houston, TX, USA)** were used as labelling proteins for the cytoplasm and membrane, respectively.

(Page **18**, Line [**347**]), **the revised objective now reads:**

To further characterize the changes in the subcellular localization of AnxA2, goat anti-mouse IgG/Cy3 **(Servicebio, Wuhan, China)** was used as the secondary antibody, and AnxA2 translocation in DF-1 cells was observed using a Ultra Acquire Ultra microscope.

L253: **The anti-AnxA2**

Response:

We appreciate the reviewer's suggestion to clarify the source of this primary antibody. This antibody was generated in our laboratory. We performed sequence homology comparison and confirmed that the chicken target protein has approximately 89.38 % amino acid identity with the immunogen for antibody preparation. On this basis, we used this homemade antibody to detect the protein in DF-1 cells. We have supplemented the above information in the Materials and Methods (Page 7, Line 134)

(Page **7**, Line [**134**]), **the revised objective now reads:**

The polyclonal anti-AnxA2 serum was prepared in our laboratory.

Comment 59:

L181 protein fractions were divided

Page **13**, Line [**245**], **the revised objective now reads:**

The cytoplasmic protein **fractions** and membrane protein **fractions** were divided into upper aqueous layers and lower oil layers, respectively.

Comment 60:

L213 Avoiding light, 3.3...

Page **15**, Line [**289**], **The revised objective now reads:**

Avoiding light, 3,3',5,5'-tetramethylbenzidine colour development solution was added.

Comment 61:

L232 Please include the source of RT, RIPA (L236), Image J (L238), L262 GraphPad

Page **16**, Line [**313**], **the revised objective now reads:**

cDNA was synthesized through reverse transcription **with reagent kit (ABclonal, Wuhan, China)**, and specific primers for AnxA2 and GAPDH were used for RT–qPCR detection (Table 1).

Page **17**, Line [**324**], **the revised objective now reads:**

Additionally, total protein was extracted from infected cells with RIPA lysis buffer **(Solarbio)**, quantified by the BCA method, and analysed via Western blotting.

Page **17**, Line [**326**], **the revised objective now reads:**

The protein bands were visualized using ECL and quantified with ImageJ software **(National Institutes of Health, USA)**.

L262 GraphPad

Response:

We sincerely appreciate your valuable comments. Supplementary information has been added at the first mention of this software, and the rest of the manuscript remains unchanged in the revised version.

Page **9**, Lines [**175-176**], **the revised objective now reads:**

, and a homology heatmap was generated using GraphPad Prism 8.0 software **(GraphPad Software, Inc., San Diego, California, USA)**.

Comment 62:

L242 Please rephrase "crawled"

Page **17**, Line [**330**], **the revised objective now reads:**

To test the interaction potential of SSB with AnxA2, DF-1 cells were inoculated in 6-well plates containing **round coverslips**, and after the cell confluence reached 80%, the DF-1 cells were incubated with SSB at 39 °C for 3 hours.

Comment 63:

L246 an Ultra (also L257)

Page **18**, Line [**348**], **the revised objective now reads:**

To further characterize the changes in the subcellular localization of AnxA2, goat anti-mouse IgG/Cy3 was used as the secondary antibody, and AnxA2 translocation in DF-1 cells was observed using **an** Ultra Acquire Ultra microscope.

Page **17**, Line [**335**], **the revised objective now reads:**

The nuclei were stained with DAPI and then imaged and analysed using **an** Ultra Acquire Ultra microscope for colocalization of SSB with AnxA2.

Comment 64:

L251 Which kit?

Page **18**, Line [**341**], **The revised objective now reads:**

, before the DF-1 cells membrane and cytoplasmic proteins were extracted using the Cell Membrane Protein Extraction Kit (Beyotime).

Comment 65:

L260 Please define SEMs

Page **18**, Line [**352**], **The revised objective now reads:**

All the data are expressed as the means ± SEMs **(Standard Error of the Mean)**.

Comment 66:

L282 of the E. coli

Page **20**, Line [**400**], **the revised objective now reads:**

The overlap PCR product results demonstrated that the amplified specific bands, corresponded to the anticipated 567 bp, confirming the successful construction of the prokaryotic expression vector pET-*ssb* **of the *E. coli***.

Comment 67:

L285 transformed with the empty

Page **21**, Line [**406**], **the revised objective now reads:**

This band was absent in the control group **transformed** with the empty vector pET-28a (+), indicating the successful expression of the recombinant protein SSB.

Comment 68:

L315 compared with that

Page **23**, Line [**441**], **the revised objective now reads:**

The RT–qPCR analysis results revealed that the AnxA2 mRNA expression level increased with increasing SSB duration **compared** with that in the control group, and the difference was statistically significant at 3 hours ($p < 0.0001$).

Comment 69:

L319 transcription

Page **23**, Line [**445**], **the revised objective now reads:**

These findings suggest that SSB may increase AnxA2 protein expression by increasing AnxA2 **transcription**.

Comment 70:

L333 Secreted bacterial proteins from pathogens are mainly adhesins, catabolic enzymes, toxins and ..

Page **23**, Line [**460**], **the revised objective now reads:**

Secreted bacterial proteins **from pathogens** are mainly adhesins, **catabolic enzymes**, toxins, and virulence-determining enzymes,

Comment 71:

L337 These genera can be abbreviated to *M.*

Page **24**, Lines [**464-466**], **the revised objective now reads:**

For example, ***M.*** *bovis* and ***M.*** *mycoides subsp. mycoides* have 55 and 44 secreted proteins, respectively, and ***M.*** *hyopneumoniae* and ***M.*** *flocculare* in swine have 62 and 26 secreted proteins, respectively.

Comment 72:

L337 subsp. does not require italicization; also L343 (the species name should not be capitalized)

Page **24**, Line [**465**], **the revised objective now reads:**

For example, *Mycoplasma bovis* and *Mycoplasma mycoides* **subsp**. *mycoides* have 55 and 44 secreted proteins, respectively,

Page **24**, Line [**471**], **the revised objective now reads:**

The secreted proteins phosphonate ABC transporter phosphonate-binding protein and BspA family leucine-rich repeat surface protein have been identified as potential molecular markers of *mycoplasma mycoides* **subsp**. *Mycoides* infection.

Comment 73:

L339 flocculare in swine have

Response:

We have carefully checked this sentence on several occasions and verified that the original text contains no grammatical or spelling errors. Fully taking the reviewer's valuable advice into full consideration, we have standardized the italic format of mycoplasma Latin names for stricter academic expression in the revised manuscript.

Page **24**, Line [**466**], **the revised objective now reads:**

, and ***M.*** *hyopneumoniae* and ***M.*** *flocculare* in swine have 62 and 26 secreted proteins, respectively.

Comment 74:

L340 Among them, the *M. bovis* secreted proteins

Page **24**, Line [**467**], **the revised objective now reads:**

Among them, the ***M. bovis*** secreted **proteins** MbovP274 and MbovP570 are immunogenic.

Comment 75:

L344 Please rephrase this sentence as the authors intended meaning was not clear to me

Page **24**, Lines [**473-477**], **the revised objective now reads:**

These findings **clearly demonstrate that mycoplasma secreted proteins exhibit species-specific characteristics in terms of distribution, abundance, and function. Not only do they serve as potential molecular diagnostic markers, but they also lay a theoretical foundation for the screening of candidate antigens and further investigation into the pathogenic mechanisms of mycoplasmas.**

Comment 76:

L347 identified SSB (or is it plural? Were more than one type of SSB identified)? Also match protein/proteins as singular or plural depending on whether SSB or SSBs is used

Page **25**, Line [**485**], **the revised objective now reads:**

, that the single-stranded DNA-binding protein **is a** secreted protein.

Comment 77:

L358 Please include locus tag or other identifier for this ABC ATP cassette as there will be multiple in *M. bovis*

Page **26**, Line [**513**], **the revised objective now reads:**

Furthermore, the ABC transporter ATP-binding protein of *Mycoplasma bovis* (**MbovP0581**) has been identified as an immunogenic secreted protein and is predicted to function as a virulence-associated factor, potentially playing a significant role in the pathogenic mechanisms of *Mycoplasma bovis*.

Comment 78:

L361 pathogenic mechanisms in this species [31].

Page **26**, Line [**515**], **the revised objective now reads:**

Furthermore, the ABC transporter ATP-binding protein of *Mycoplasma bovis* has been identified as an immunogenic secreted protein and is predicted to function as a virulence-associated factor, potentially playing a significant role in the pathogenic mechanisms **in this species.**

Comment 79:

L361 The sentence that starts "In this study" is disconnected from the previous one. Please modify the transition or use a new paragraph

Response:

Thank you for pointing out this logical discontinuity. As suggested, it has been segmented into new paragraphs. The revised content has been recorded in the marked manuscript. This sentence starting with "In this study" is **located at Page 26, Line 517.**

Comment 80:

L362 for SSBs.

Page **26**, Line [**518**], **the revised objective now reads:**

In this study, LC–MS/MS analysis revealed a high coverage rate of polypeptide sequences for **SSBs**.

Comment 81:

L367 characterization in this pathogen.

Page **27**, Line [**522**], **the revised objective now reads:**

our results further substantiate the secretion of the SSB protein in *M. synoviae* strain WVU1853 and provide novel insights into its functional characterization in **this pathogen**.

Comment 82:

L369 receptor for certain *Mycoplasma*

Page **27**, Line [**525**], **The revised objective now reads:**

The extracellular matrix serves as a barrier against *Mycoplasma*, but plasminogen acts as a receptor for **certain** *Mycoplasma* adhesins, and its activation leads to matrix degradation, weakening tissue defence and facilitating *Mycoplasma* invasion.

Comment 83:

L371 spelling of Mycoplasma

Page **27**, Line [**527**], **The revised objective now reads:**

The extracellular matrix serves as a barrier against *Mycoplasma*, but plasminogen acts as a receptor for *Mycoplasma* adhesins, and its activation leads to matrix degradation, weakening tissue defence and facilitating ***Mycoplasma*** invasion.

Comment 84:

L372 *M. hyopneumoniae* with the

Page **27**, Line [**528**], **the revised objective now reads:**

Fibronectin facilitates the interaction of ***M. hyopneumoniae*** with the epithelial cell integrin β1, aiding in internalization.

Comment 85:

L380 that Fn and tissue ; L381 Fn

Page **29**, Lines [**573-574**], **the revised objective now reads:**

Previous studies have demonstrated that **Fn** and tissue plasminogen activator (tPA) facilitate the conversion of **Fn** into active fibrinolytic enzymes by utilizing the AnxA2 heterotetramer protein complex as a coreceptor, thereby increasing fibrinolysis.

Comment 86:

L384 Please include a reference for this fact

We have supplemented a relevant reference to support this statement in the revised manuscript at **Page 38, Line [789-792]**, and the citation has been marked in the text with track changes for easy identification.

Comment 87:

L393 by assisting tPA in converting Plg

Page **30**, Line [**587**], **the revised objective now reads:**

Nonetheless, further research is necessary to determine whether AnxA2 enhances *M. synoviae* infection by assisting **tPA** in converting **Plg** to plasmin.

Comment 88:

L399/400 Fn Plg

Page **31**, Lines [**602-603**], **the revised objective now reads:**

Additionally, SSB protein interacts with **Plg** and **Fn**, binds to DF-1 cell membrane proteins, and adheres to the cell surface.

Comment 89:

L549 Should Mycoplasma be capitalized (depends how it was published)

Page **38**, Line [**798**], **the revised objective now reads:**

Wang YP, Wang G, Hong X, Zhao J, Wu D, Chen L, Liu XL, Kong DY, Huang QC,

Xing JL, Wang N, Zhao YL. 2023. Downregulated mitochondrial transcription factor A enhances ***Mycoplasma*** infection to promote the metastasis of hepatocellular carcinoma. Cancer Sci 114:1464-1478

Comment 90:

L574 The restriction enzyme sites are underlined, the mutated site is bolded and italicized

Page **40**, Lines [**822-823**], **the revised objective now reads:**

The Underlined: The restriction enzyme sites **are underlined, the mutated site is bolded and italicized.**

Comment 91:

L577 supernatant

Page **40**, Line [**825**], **the revised objective now reads:**

TABLE 2 Secreted proteins localized to the **supernatant**

Comment 92:

Table 3 colony number Blank control

On Page **42, Table 3, The revised objective now reads:**

Colony number Blank control

Comment 93:

L585 Transmembrane region prediction results for SSB

In Supplemental Legends FIG S3, Page **1**, Lines [**11-12**], **the revised objective now reads:**

Transmembrane region prediction results for SSB.

Comment 94:

Fig 2 and 3; kDa rather than KDa (also additional file 1

Response:

We appreciate the reviewer's careful correction of the molecular weight unit format. We have uniformly revised all incorrect "KDa" notations to the standard form kDa throughout FIG 2, FIG 3 and Supplemental Figures FIG S4 c, and double-checked all figure legends and legends in the supplementary file to ensure consistent formatting.

Comment 95:

L599 Fn to SSB was detected

Page **45**, Line [**852**], **the revised objective now reads:**

The binding of Fn to **SSB was** detected by ELISAs.

Reviewer #2

This study reports a secreted protein of M. synoviae and suggests a role for this protein in pathogenicity. The study is of interest to the readership of the journal and especially to mycoplasmologists and veterinarians. The authors claim that the protein identified is a single stranded binding protein but do not provide functional evidence about the preferred binding of the protein to single stranded DNA over double stranded DNA. They make a reference to the paper Rebello Couto et al. 2008, but this paper does not report the functional confirmation of a secreted SSB in M. synoviae. Therefore, they either rephrase or include additional functionally experiments. In case the protein sequence shows very high similarity to an SSBs for which activity was experimentally confirmed, this should be stated. I suggest the authors have a look at the presence and diversity of the encoding gene in other M. synoviae and Mycoplasma genomes and add these data to the manuscript to get an idea about the conservation and prevalence of the gene in other strains of the species and in other species. This task can easily be executed and it will add value to the paper.

The methods section needs to be expanded, since it does not provide enough details that allow fellow scientist to replicate experiments in related pathogens. The IACUC number and IACUC committee name for the rabbit immunizations must be provided and the experiment should be described according to ARRIVE guidelines. If the cloning of the antigen in E. coli included codon optimization, which I am only assuming, it should be mentioned. The authors refer to harvesting the cells in the log phase but did not report how the log phase was determined. They have to add how they determined the log phase for their pathogen.

Given the nature of the identified 'secreted' proteins, a critical discussion about pitfalls related to the methods applied should be added. Being devoid of a cell wall means that any little rupture of the cell membrane due to forces exposed by the protocols (mechanical or chemical) would result in leakage of cytoplasmic content into the surrounding media and this should be discussed. Moreover, they should discuss the fact that a signal sequence that is often associated with secreted proteins is not present in

the gene sequence encoding their 'secreted' protein. What is the model that enables a secreted protein to act as adhesion in M. synoviae?

The authors claim that the 'Single-Stranded DNA-Binding Protein' investigated in this study is an important virulence factor but do not prove this statement experimentally. This study claims involvement in pathogenicity, which might well, but the study does not at all provide evidence that this factor is an important virulence factor. I advice the authors to study Falkow's molecular postulates and to weigh their data accordingly.

Response:

Thank you very much for your thorough and constructive comments on our manuscript. we greatly appreciate your careful evaluation, which includes a general overview of the manuscript's limitations followed by detailed point-by-point suggestions, among which several supplementary scientific issues are raised that were not covered in the initial general assessment. All your valuable criticisms have been fully addressed, and corresponding revisions and supplementary analyses have been completed in the revised manuscript. Modifications are highlighted in red throughout the text, and our detailed responses to each comment are presented below.

Responses to detailed comments derived from the general evaluation

Comment 1:

This study reports a secreted protein of *M. synoviae* and suggests a role for this protein in pathogenicity. The study is of interest to the readership of the journal and especially to mycoplasmologists and veterinarians. The authors claim that the protein identified is a single stranded binding protein but do not provide functional evidence about the preferred binding of the protein to single stranded DNA over double stranded DNA. They make a reference to the paper Rebello Couto et al. 2008, but this paper does not report the functional confirmation of a secreted SSB in *M. synoviae.* Therefore, they either rephrase or include additional functionally experiments. In case the protein sequence shows very high similarity to an SSBs for which activity was experimentally confirmed, this should be stated.

Response:

Thanks for your constructive suggestions. The protein was initially annotated as "single -stranded DNA-binding protein" in the NCBI Refseq database (accession: WP_011283670). This name assignment is based on **conserved domain (CDD) prediction** via the NCBI SPARCLE system, which identifies the protein as a member of the SSB (single-stranded DNA-binding) protein family. This classification is supported by the review "The tale of SSB" (Bianco, 2017), which demonstrates that SSB family proteins are highly conserved across prokaryotes and typically function in ssDNA binding.

However, our current study focuses on the virulence-related roles of this protein in *Mycoplasma synoviae*, and we have not performed direct biochemical assays to confirm its ssDNA-binding activity. Therefore, following the reviewer's suggestion, we have revised the protein name to *M. synoviae* SSB protein, using the official gene symbol (SSB) to avoid implying unvalidated functional data in our work.

Page **1**, Lines [**1-2**], **the revised objective now reads:**

A Novel Secreted Protein of *Mycoplasma synoviae*, **SSB**, is a Critical Adhesin Factor that Promotes the Enrichment of Annexin A2 at the Cell Membrane

Comment 2:

I suggest the authors have a look at the presence and diversity of the encoding gene in other *M. synoviae* and Mycoplasma genomes and add these data to the manuscript to get an idea about the conservation and prevalence of the gene in other strains of the species and in other species. This task can easily be executed and it will add value to the paper.

Response:

We highly appreciate this valuable suggestion. We have now performed **homology analysis and phylogenetic tree analysis to** explore the distribution, genetic diversity and sequence conservation of the gene across different *M. synoviae* strains and other mycoplasma species. The relevant results have been supplemented in the revised

manuscript, which helps to illustrate the prevalence and conservation of the target gene.

Pages **9-10**, Lines [**164-182**], **the revised objective now reads:**

Sequence alignment, phylogenetic tree construction and conserved domain analysis

Using the DNASTAR software (DNASTAR, Inc., Madison, Wisconsin, USA), the SSB amino acid sequences (AKB11260.1) of the *M. synoviae* WVU1853 strain were compared with those of *M. synoviae* HN01 (WP_154221707.1), *M. synoviae* H (AQU48076.1), *M. synoviae* 53 (WP_011283670.1), *M. synoviae* 5-9 (UBM43515.1), *M. synoviae* A4 (UBY00073.1), *M. synoviae* G3 (UBX99132.1), *Mycoplasma gallisepticum* (ADC30831.1), *M. pneumoniae* (XBW34012.1), *M. mycoides* subsp. *mycoides* (AMK55955.1), *M. agalactiae* (UUM25159.1), *M. anserisalpingitidis* (QDY88381.1), *Mesomycoplasma hyorhinis* (*M. hyorhinis*) (QEA02011.1), *M. hyopneumoniae* (WP_404924450.1), *M. hyopneumoniae* 7448 (AAZ53668.1), and *M. bovis* HB0801 (WP_013954802.1) from the NCBI database was performed, and a homology heatmap was generated using GraphPad Prism 8.0 software (GraphPad Software, Inc., San Diego, California, USA). Phylogenetic analysis was conducted using the MEGA 8.0 software (Institute of Molecular Evolutionary Genetics, Pennsylvania, USA). The phylogenetic tree was built via the Neighbor-Joining (NJ) method with Poisson correction model. Bootstrap analysis with 1,000 replicates was performed to evaluate the reliability of each branch. In addition, the SSB amino acid sequence was analyzed using the NCBI Conserved Domain Database (CDD). The predicted domain structure was then compared with the reported domain features of *E. coli* and *Bacillus subtilis* SSB from published literatures.

Pages **19-20**, Lines [**375-388**], **the revised objective now reads:**

Sequence similarity, phylogenetic analysis, and conserved domain identification

Based on the homology heatmap (Fig 1a) and the phylogenetic tree (Fig 1b), we analyzed the genetic and evolutionary relationships between *M. synoviae* strain

WVU1853 and its closely related strains. Sequence homology analysis revealed that the SSB amino acid sequences of *M. synoviae* strain WVU1853 exhibited high homology (over 98%) with those of *M. synoviae* strains HN01, H, 53, A4 and G3. Homology with strains such as *M. gallisepticum* and *M. pneumoniae* was low (below 25%). Phylogenetic tree analysis showed that *M. synoviae* WVU1853 clustered with strains HN01, H, 53, and 5-9 in the same evolutionary branch, exhibiting the closest phylogenetic relationship. It also showed a relatively high degree of relatedness with *M. synoviae* G3 and A4, while exhibiting the lowest relatedness with genera *Mesomycoplasma* and *Mycoplasmoides*. These findings indicate that the SSB highly conserved within the *M. synoviae* species, yet genetic differentiation still exists among different strains of the same species, while SSB sequences across genera show significant differentiation.

.

Comment 3:

The methods section needs to be expanded, since it does not provide enough details that allow fellow scientist to replicate experiments in related pathogens.

Response:

We fully agree with this comment. We have comprehensively supplemented detailed experimental information across the entire Materials and Methods section (Page 6-18, Lines 118-350).

Uniform supplementary experimental parameters have been added to each assay subsection, including centrifugation speed, reagent volume, incubation duration, reaction temperature, primary and secondary antibody dilution ratios, as well as standardized replicate and negative control setting.

For example:

Pages **7-8**, Lines [**140-144**], **the revised objective now reads:**

The supernatant was collected by centrifugation at 12,000×g for 45 min at 4 °C (Beckman Coulter, Inc., Brea, CA, USA) **to remove intact mycoplasma cells**. Proteins in the supernatant were **kept on ice throughout enrichment** using a tangential flow

filtration membrane package (Sartorious, Germany) with a molecular weight cut-off of 10 kDa **at a pump speed of 60 rpm. After the supernatant was concentrated to approximately 50 mL,**

Comment 4:

The IACUC number and IACUC committee name for the rabbit immunizations must be provided and the experiment should be described according to ARRIVE guidelines.

Response:

The IACUC approval number and full name of the animal ethics committee have been supplemented in the ETHICS STATEMENTS, and the description of rabbit immunization experiments has been revised in accordance with the ARRIVE guidelines.

Pages **11-12**, Lines [**216-221**], **the revised objective now reads:**

(i) Ethics statements

Animal housing and care complied with institutional laboratory animal guidelines. All animal experiments were performed, and all experimental data and procedures reported, in accordance with the ARRIVE guidelines 2.0 (2020). All experimental procedures were reviewed and approved by the Institutional Animal Care and Use Committee (IACUC) of Gansu Agricultural University (Approval No: GSAU-Eth-VMC-2024-087).

Comment 5:

If the cloning of the antigen in *E. coli* included codon optimization, which I am only assuming, it should be mentioned.

Response:

We have performed codon optimization only for internal stop codons within the gene sequence, and this modification has been clearly stated in the revised manuscript. No other codon optimization was conducted during gene cloning and prokaryotic expression in *Escherichia coli*.

Page **10**, Lines [**185-188**], **the revised objective now reads:**

The *ssb* sequence contains a TGA codon at 163-165 bp. This codon encodes tryptophan in mycoplasma, whereas in *E. coli* it functions as a stop codon. To achieve site-directed codon mutations within the gene and successfully express SSB, overlap PCR was used to amplify the *ssb* and **optimized** the 165 bp site,

Comment 6:

The authors refer to harvesting the cells in the log phase but did not report how the log phase was determined. They have to add how they determined the log phase for their pathogen.

Response:

The logarithmic growth phase was determined using the growth curve previously established in our laboratory via colony forming units (CFU) (Yunhai Zhao, 2024; Tingwen Li et al.,2026). Cells were collected at the corresponding time point. This information together with citation has been added to the revised manuscript.

Page **7**, Lines [**138-139**], **the revised objective now reads:**

The timing of the logarithmic growth phase was defined by the standard growth curve of the *M. synoviae* WVU1853 strain.

Comment 7:

Given the nature of the identified 'secreted' proteins, a critical discussion about pitfalls related to the methods applied should be added. Being devoid of a cell wall means that any little rupture of the cell membrane due to forces exposed by the protocols (mechanical or chemical) would result in leakage of cytoplasmic content into the surrounding media and this should be discussed.

Response:

We sincerely appreciate this critical and valuable comment. We fully recognize that the absence of a cell wall in *M. synoviae* and the plasma membrane is extremely fragile. Centrifugation, filtration, and tangential flow filtration circulation generate mechanical shear stress that may trigger subtle membrane rupture, releasing abundant cytoplasmic

proteins and contaminating the secreted protein fraction. We have supplemented a comprehensive critical discussion of all methodological pitfalls and corresponding control strategies in the revised Discussion section as requested.

Pages **24-26**, Lines [**479-503**], **the revised objective now reads:**

Distinguishing between actively secreted proteins and intracellular proteins released by bacterial lysis has long been a challenge in mycoplasma secretome research. In 2012, Rebollo Couto et al. successfully cultured *M. synoviae* strain 53 using a modified protein-free Frey medium, **concentrated the supernatant using an ultrafiltration system, and then precipitated extracellular proteins via TCA-acetone precipitation. They confirmed, using two-dimensional (2-DE) electrophoresis, that the** single-stranded DNA-binding protein is a secreted protein. In the present study, we supplemented mycoplasma broth with 10% swine serum to support normal growth. **Whole cells were first removed by low-speed centrifugation, and supernatant proteins were then enriched via TFF for mass spectrometry identification. However, during the TFF filtration process, the high-speed circulation of fluid between the feed pump and the membrane module generates significant shear forces, which can easily cause membrane rupture in mycoplasma, leading to the leakage of cytosolic proteins. To minimize protein operation-induced bacterial lysis as much as possible, all procedures in this study were performed in an ice bath, and transmembrane pressure and circulation flow rates were strictly minimized. Additionally, drawing on established quality control protocols for the *M. bovis* secretome, colony-forming unit (CFU) counts were used to quantify the number of viable *M. synoviae* before and after centrifugation, thereby assessing the risk of cell membrane rupture induced by mechanical shear forces. The results showed no significant decrease in viable cell counts after completion of the entire pretreatment process. Furthermore, CFU testing of the supernatant after centrifugation demonstrated that by controlling the number of residual live bacteria in the supernatant, the extent of cytoplasmic protein leakage was kept at a low level. Even with the risk of lysis reduced through multiple optimizations,**

trace amounts of mycoplasma rupture caused by fluid shear cannot be completely eliminated, which is an unavoidable limitation of this method.

Comment 8:

Moreover, they should discuss the fact that a signal sequence that is often associated with secreted proteins is not present in the gene sequence encoding their 'secreted' protein. What is the model that enables a secreted protein to act as adhesion in *M. synoviae*?

Response:

We sincerely appreciate your constructive suggestion. We have added detailed discussion and proposed a model to address this question in the revised Discussion section.

Pages **27-28**, Lines [**535-554**], **the revised objective now reads:**

In **this** study, **bioinformatics predictions indicated that SSB lacks the N-terminal signal peptide characteristic of classical secretory proteins, suggesting that the protein is not transported via the classical secretory pathway but is primarily released into the extracellular space through the non-classical secretory pathway. Consistent with subcellular fractionation and immune-gold labeling results, SSB was not detected on the membrane of *M. synoviae* WVU1853, ruling out the membrane-anchored form of SSB on the *M. synoviae* surface. Subsequently, we found that moonlighting protein SSBs can specifically bind** to **the host cell membrane proteins and extracellular matrix proteins** Plg and Fn, with binding increasing as SSB levels increase**.** These results confirm that SSB mediates the adhesion of *M. synoviae* to DF-1 cells and suggest that the SSB protein may be closely associated with the internalization and invasion of *Mycoplasmopsis synoviae*.

Comment 9:

The authors claim that the 'Single-Stranded DNA-Binding Protein' investigated in this study is an important virulence factor but do not prove this statement experimentally.

This study claims involvement in pathogenicity, which might well, but the study does not at all provide evidence that this factor is an important virulence factor. I advice the authors to study Falkow's molecular postulates and to weigh their data accordingly.

Response:

We sincerely appreciate this rigorous and insightful comment, as well as the valuable reminder of Falkow's molecular postulates for evaluating virulence factors.

We fully acknowledge that the current work lacks gene knockout/functional complementation in *M. synoviae* to directly satisfy all criteria of Falkow's molecular postulates, which is the gold standard to definitively confirm a protein as a key virulence factor. Given the technical limitations of genetic manipulation in *M. synoviae*, stable gene deletion or expression strains are extremely difficult to construct at present, and we clearly recognize this limitation of our study. Accordingly, we have revised our wording throughout the manuscript to avoid overstating that SSB is an important virulence factor. We use moderate and evidence-based descriptions: SSB may serve as a potential virulence factor contributing to the pathogenic process of *M. synoviae*. We have explicitly discussed that limitation and the gap to fully meet Falkow's postulates in the revised Discussion section.

Page **2**, Line [**32**], **the revised objective now reads:**

Bioinformatic analysis of SSBs revealed that they lack a transmembrane domain and the characteristic features of a cleavable signal peptide and are **potential** virulence factors.

Pages **30**, Lines [**588-597**], **the revised objective now reads:**

According to Falkow's molecular postulates, to definitively identify a protein as a key virulence factor, gene knockout, gene restoration, and in *vivo* infection model validation must be completed. Due to the limitations of the current experimental conditions, *ssb* knockout and rescue strains have not yet been constructed, nor have in *vivo* infection experiments in poultry been conducted. At present, relevant functional results have been obtained only through in *vitro* experiments; the existing evidence can only support functional inferences at the in *vitro* level and

does not fully meet all the criteria of the Falkow molecular postulates. In future studies, a series of experiments combining genetic manipulation with in *vivo* infection in poultry can be conducted to further substantiate the virulence role of this protein.

Responses to additional new comments not mentioned in the general evaluation

Comment 1:

Line 24-26: I suggest to add the eggshell abnormalities.

Response:

Thank you for your valuable suggestion. We have supplemented the relevant information in the revised manuscript, and all revisions are marked with track changes.

Page **2**, Line [**25**], **the revised objective now reads:**

Mycoplasma synoviae (*M. synoviae, MS*) is an important pathogen in poultry, that primarily causes synovitis, tenosynovitis, **eggshell abnormalities**, and respiratory diseases, resulting in significant economic losses to the poultry industry worldwide.

Comment 2:

Line 63: It is new to me that *M. synoviae* is the smallest prokaryotic organism, provide a reference if this is really the case.

Response:

Thanks for pointing out this inaccurate description. We have revised the inappropriate statement regarding *M. synoviae* and added supporting references; all edits are tracked in the manuscript.

Page **4**, Line [**64**], **the revised objective now reads:**

It was first reported as an unknown pathogen in the United States in 1954 and is **one of** the smallest **self-replicating** prokaryotic **organisms** found to date.

Comment 3:

Line 91: Write ..can increase ...

Response:

Thank you for spotting this grammatical mistake. We have corrected it, and the modification is marked with track changes.

Page **5**, Line [**94**], **the revised objective now reads:**

In recent years, it has been shown that SSBP1 can **increase** p53 phosphorylation and its nuclear accumulation through interaction with DNA-dependent protein kinases, which subsequently drives ferroptosis in podocytes.

Comment 4:

Line 169-171: Be specific for example in terms of g-force, amounts of liquids and dilutions of antibodies.

Response:

Thank you for this valuable suggestion. We have supplemented specific experimental parameters including centrifugal g-force, liquid volumes and antibody dilution ratios in the revised manuscript's.

Page **12**, Lines [**232-238**], **the revised objective now reads:**

One millilitre *M. synoviae* culture was centrifuged **with 8,000×g for 10 min in 4 ℃** and resuspended in PBS **with 200 μL**, and **30 μL** of the mixture was collected, spread evenly on a slide, and left to dry naturally. The samples were fixed 4% paraformaldehyde, incubated with primary antibodies (anti-SSB serum; preimmunization serum as negative control and anti-*M. synoviae* serum as a positive control; **all sera were diluted 1:1,000**), incubated with goat anti-rabbit IgG/FITC antibodies (**diluted** ratio as **1: 500**) as secondary antibodies, and finally observed under a fluorescence microscope (ZEISS, Germany).

Comment 5:

Line 189: Write out fibronectin and plasminogen before you abbreviate

Response:

Thank you for the reminder. We have supplemented the full name prior to the

corresponding abbreviation at its first occurrence in the revised manuscript, and all revisions are marked with track changes.

Page **13**, Line [**254**], **the revised objective now reads:**

Binding of SSB to chicken **Plasminogen** (Plg) and **Fibronectin** (Fn)

Comment 6:

Line 319: Write 'transcription'

Response:

Thank you for noticing this error. We have revised the misspelled word to "transcription" in the manuscript, and all edits are tracked.

Page **23**, Line [**445**], **the revised objective now reads:**

These finding suggest that SSB may increase AnxA2 protein expression by increasing AnxA2 **transcription.**

Comment 7:

The mass spectrometry data should be deposited as additional file or uploaded to a database so that it can be accessed by the readership.

Response:

All complete mass spectrometry-related materials have been compiled and uploaded as Supplemental material (Table S1, Table S2, FIG S1). The uploaded contents include: 1) Excel spreadsheets containing full peptide identification information and protein results; 2) representative TIC (total ion current) plots of each sample. Raw original data stored in Excel files have been clearly indicated in the data availability section on Page 31, Lines 606. All these files are publicly available alongside the manuscript, allowing readers to access and reanalyze the proteomic data.

Comment 8:

Line 599: Change to: The binding of Fn to SSB was detected by ELISAs

Response:

We appreciate your careful correction. The grammatical error has been fixed in the revised manuscript, and the revision is highlighted via track changes.

Page **45**, Line [**852**], **the revised objective now reads:**

The binding of Fn to **SSB was** detected by ELISAs.

Comment 9:

I suggest to move Figure 1 to the supplementary material, since it displays only negative in silico data.

Response:

Thanks for your valuable suggestion. We have relocated Figure 1 to the supplementary material as recommended. This figure is now presented in Supplemental Figures FIG S3, and all in-text citations to this figure have been renumbered accordingly.

Reviewer #3:

The paper titled "A Novel Secreted Protein of *Mycoplasma synoviae*, Single-Stranded DNA-Binding Protein, is a Critical Adhesion Factor that Promotes the Enrichment of Annexin A2 at the Cell Membrane," following the screening of *Mycoplasma synoviae* secretory proteins, the authors focused on the gene cloning, protein expression and purification, and antibody preparation of the single-stranded DNA-binding protein (SSB) and subsequently investigated its interaction with host cells. These findings provide a new theoretical perspective for elucidating the pathogenic mechanism of *Mycoplasma synoviae* and offer potential molecular targets for the prevention of *Mycoplasma synoviae* infection. The experimental design has clear scientific objectives, and the overall experimental content demonstrates a certain degree of scientific rigor and completeness; however, some details still require refinement. The specific review comments are as follows:

Areas for Improvement and Suggestions for Revision

Response:

We sincerely appreciate the reviewer for carefully reading our manuscript and delivering this objective and comprehensive evaluation. We are grateful for your positive recognition of our research objective, experimental integrity, and scientific significance of SSB in *Mycoplasma synoviae*. Furthermore, we fully acknowledge that several experimental details need further optimization, and we will earnestly revise and respond to every specific comment point by point in the revised manuscript.

Comment 1:

The culture conditions for *Mycoplasma synoviae* have not yet been clearly defined, including culture temperature and the required gas environment, which still need to be further clarified.

Response:

Thank you for pointing out this missing information. We have supplemented the complete culture conditions for *M. synoviae* WVU1853 in Materials.

Page **7**, Line [**125**], **the revised objective now reads:**

All cultures were incubated at 37°C in a 5% CO_2 incubator.

Comment 2:

The virulence factor predictions have been performed only for single–stranded DNA binding protein among the 13 identified secreted proteins, which fails to highlight the overall value of the screening results. It is recommended that annotations of virulence factor predictions for all secreted proteins in the screening be added to Table 2.

Response:

We fully agree with this suggestion. We have completed virulence factor prediction for all 13 identified secreted proteins and supplemented the corresponding annotation information in Table 2 in the revised manuscript so as to comprehensively display the biological value of our secretome screening data.

Pages **40-42**, Lines [**825-826**], **the revised objective now reads:**

TABLE 2 Secreted proteins localized to the supernatants

**Accession Number**	**Protein names**	**Prediction Results**
AKB11101.1	deoxyribonuclease	Virulent
AKB11382.1	Elongation factor Tu	Non-virulent
AKB11096.1	VY93_01090 rRNA methyltransferase	Virulent
AKB10884.1	ABC transporter substrate-binding protein	Virulent
AKB11260.1	single-stranded DNA-binding protein	Virulent
AKB11161.1	DNA helicase	Virulent
AKB11174.1	histidyl-tRNA synthetase	Non-virulent
AKB10936.1	hypothetical protein VY93_00850	Virulent
AKB10998.1	lipoprotein	Virulent

AKB10971.1	peptide ABC transporter ATP-binding protein	Non-virulent
AKB10972.1	hypothetical protein VY93_01090	Virulent
AKB11015.1	lipoprotein	Virulent
AKB11330.1	ABC transporter permease	Virulent

Accession number of proteins and protein names are derived from the NCBI database.

Comment 3: Standardize terminology for protein molecular weight units to eliminate inconsistencies and ensure consistent use of standard terms such as "kDa" throughout the manuscript.

Response:

Thank you for this careful reminder. We have systematically checked and unified all protein molecular weight notations in the manuscript, adopting the standard unit “kDa” throughout the text to eliminate inconsistent terminology. All relevant revisions are marked in red.

Comment 4:

Number of replicates not specified: Some experiments in the manuscript do not specify the number of replicates (e.g., three independent replicates). The reliability of the experimental results cannot be guaranteed. It is recommended to supplement the manuscript with the number of replicates used for the relevant experiments.

Response:

We highly appreciate the reviewer’s careful reminder regarding experimental replicates. We have supplemented the number of biological and technical replicates for all related experiments throughout the revised manuscript as requested.

Page **14**, Lines [**267-268**], **the revised objective now reads:**

SSB binding to Plg and Fn was assessed by using optical density (OD_{450}) absorbance measurements, **and three independent replicates were performed**.

Page **15**, Line [**292**], **the revised objective now reads:**

BSA was used as a negative control**, and all assays were performed in three independent replicates.**

Comment 5: Although the manuscript provides RT-qPCR results for Annexin A2 gene expression levels, the experimental section lacks a description of key RT-qPCR parameters. The manuscript should detail the RT-qPCR reaction system and amplification protocols.

Response:

Thank you for pointing out this defect. We have supplemented the full RT-qPCR reaction system and amplification procedure in the Methods part, including reaction volume, reagent dosage, temperature setting and cycle parameters. All added experimental information is marked in red.

Pages **16-17**, Lines [**315-322**], **the revised objective now reads:**

The RT–qPCR setup system included 20 µL reaction with SYBR Green Master Mix **10 µL**, cDNA **1 µL**, **upstream and downstream** primers **0.2 µL, respectively**, and RNase-free water **supplemented up to 20 µL. qPCR was performed on a LightCycler 96 real-time PCR system using SYBR Green qPCR Master Mix. The thermal cycling program was set as follows: initial denaturation at 95 °C for 30 s, followed by 40 cycles of denaturation at 95 °C for 10 s and combined annealing-extension at 60 °C for 30 s. All RT-qPCR assays were performed with three independent biological replicates, and each biological replicate contained three technical replicates.**

Comment 6: Regarding the data results in the manuscript, significant differences are currently indicated only by the symbols "*" or "**" without specifying statistical thresholds. Please supplement the statistical analysis section with an explanation of the P-value criteria and thresholds corresponding to each significance level.

Response:

Thank you for pointing out this deficiency. We have added clear descriptions of p-value

cut-offs corresponding to * and ** in the statistical analysis part: No significant difference (ns) represents p⩾0.05, * represents $p < 0.05$, ** represents $p < 0.01$, and *** represents $p < 0.001$. Corresponding supplementary notes were also to each figure legend. All modifications are marked in red.

Page **18**, Lines [**355-356**], **the revised objective now reads:**

Differences between two groups were analysed using a t-test. **No significant difference (ns), *p* ≥0.05,** *$P < 0.05$**, **$P < 0.01$, ***$P < 0.001$.**

Comment 7:

The article title is too long. Please simplify it.

Response:

We have revised and shortened the article title to a more concise version as suggested by the reviewer, and the modified title is shown below:

Page **1**, Line [**1**], **the revised objective now reads:**

A Secreted Protein of *Mycoplasmopsis synoviae*, SSB, is a Critical Adhesion Factor that Promotes the Enrichment of Annexin A2 at the Cell Membrane

Comment 8:

The language requires further revision.

Response:

We fully agree with this comment. We have invited a professional English language editing service to thoroughly polish the entire manuscript. all grammatical errors, awkward expressions and non-academic descriptions have been revised. Besides, we rechecked the logic and fluency of each paragraph to meet the standard academic writing requirements of this journal. All linguistic revisions are highlighted in red in the revised manuscript.

Re: Spectrum00858-26R1 (**A Secreted Protein of *Mycoplasmopsis synoviae*, SSB, is a Critical Adhesion Factor that Promotes the Enrichment of Annexin A2 at the Cell Membrane**)

Dear Dr. Shijun Bao:

Your manuscript has been accepted, and I am forwarding it to the ASM production staff for publication. Your paper will first be checked to make sure all elements meet the technical requirements. ASM staff will contact you if anything needs to be revised before copyediting and production can begin. Otherwise, you will be notified when your proofs are ready to be viewed.

Data Availability: ASM policy requires that data be available to the public upon online posting of the article, so please verify all links to sequence records, if present, and make sure that each number retrieves the full record of the data. If a new accession number is not linked or a link is broken, provide production staff with the correct URL for the record. If the accession numbers for new data are not publicly accessible before the expected online posting of the article, publication may be delayed; please contact ASM production staff immediately with the expected release date.

Publication Fees: For information on publication fees and which article types have charges, please visit ourwebsite. We have partnered with Copyright Clearance Center (CCC) to collect author charges. If fees apply to your paper, you will receive a message from no-reply@copyright.com with further instructions. For questions related to paying charges through RightsLink, please contact CCC at ASM_Support@copyright.com or toll free at +1-877-622-5543. CCC makes every attempt to respond to all emails within 24 hours.

ASM Membership: Corresponding authors may join or renew ASM membership to obtain discounts on publication fees. Need to upgrade your membership level? Please contact Customer Service at Service@asmusa.org.

Embargo Policy: A press release may be issued as soon as the manuscript is posted on theSpectrum Latest Articles webpage. The corresponding author will receive an email with the subject line "ASM Journals Author Services Notification" when the article is available online.

Cover Image Submissions: If you would like to submit a potential Cover Image, please email a file and a short legend to spectrum@asmusa.org. Please note that we can only consider images that (i) the authors created or own and (ii) have not been previously published. By submitting, you agree that the image can be used under the same terms as the published article. Image File requirements: TIF/EPS, 7.5 inches wide by 8.25 inches tall (at least 2,250 pixels wide by 2,475 pixels tall), minimum 300 dpi resolution (600 dpi preferred), RGB, and no figure elements, e.g., arrows or panel labels. The legend should be a short description of the image, 1-2 sentences recommended. Please use the ASM Journals Cover Image Sizing Tool (https://journals.asm.org/pb-assets/html/asm-cover-sizing-tool.html) to ensure that your proposed cover image meets our size requirements.

The ASM Journals program strives for constant improvement in our submission and publication process. Please tell us how we can improve your experience by taking this quick Author Survey.

Thank you for submitting your paper to Spectrum.

Sincerely,
John Atack
Editor
Microbiology Spectrum
