# Supplementary material for: A secreted protein of Mycoplasmopsis synoviae, SSB, is a critical adhesion factor that promotes the enrichment of Annexin A2 at the cell membrane
Source: Microbiol Spectr. 2026 Aug 27;14(10):e00858-26. doi: 10.1128/spectrum.00858-26 (PMC13688872; doi:10.1128/spectrum.00858-26)
Supplement: Supplementary file 3 [file spectrum.00858-26-s0002.docx]

**FIG S1.** Raw total ion current (TIC) chromatograms of LC-MS/MS qualitative proteomics.

The unprocessed original spectral profiles represent the raw data basis for all protein identification results in this study. This figure is provided for data traceability and was not independently discusses in the main text.

**FIG S2.** Polyclonal anti-*M. bovis* Grpe serum cross-reactivity against *M. synoviae*.

M: Protein Marker, Lane 1: *M. bovis*; Lane 2: *M. synoviae* WVU1853.

**FIG S3.** Bioinformatics analysis of SSB.

(a) Signal peptide prediction of the candidate protein SSB. (b) Transmembrane region prediction results for SSB. (c) Validation of the toxicity prediction of the candidate protein SSB.

**FIG S4.** Cloning and purification of *M. synoviae* SSB.

(a) Lane M: DNA Marker; Lane 1: 1-173bp; Lane 2: 154-567bp; Lane3: full length *ssb*. (b) Lane M: DNA Marker; Lane 1: pET-28a (+) empty vector; Lane 2: double digestion reaction with *Bam*H I and *Xho* I restriction enzymes was used to verify the recombinant of *ssb*. (c) M: Prestained protein molecular weight marker; Lane 1: pET-28a (+) empty vector; Lane 2: Induced whole bacterial protein; Lane 3: Induced supernatant protein; Lane 4: Induced sediment protein; Lane 5: Purified recombinant protein.

**Table S1. Full peptide identification raw data**

This Excel contains complete peptide identification information results, including confidence, peptide sequence details, modification annotations, and quantitative abundance data.

**Table S2. *MS* WVU1853 specific protein identification results**

This Excel contains the final protein matched to the *MS* WVU1853 strain genome, with GO (Gene Ontology) functional annotation and quantitative abundance data. All protein identifications were filtered at a false discovery rate (FDR) of ＜0.01 at both peptide and protein levels.
