# Supplementary material for: A secreted protein of Mycoplasmopsis synoviae, SSB, is a critical adhesion factor that promotes the enrichment of Annexin A2 at the cell membrane
Source: Microbiol Spectr. 2026 Aug 27;14(10):e00858-26. doi: 10.1128/spectrum.00858-26 (PMC13688872; doi:10.1128/spectrum.00858-26)
Supplement: Supplementary file 4 [file spectrum.00858-26-s0001.docx]

**FIG S1**

**FIG S2**

**FIG S3**

**FIG S4**
